# Quantifying the effects of practicing a semantic task according to subclinical schizotypy

**DOI:** 10.1038/s41598-024-53468-4

**Published:** 2024-02-05

**Authors:** Mingyi Diao, Ilya Demchenko, Gifty Asare, Yelin Chen, J. Bruno Debruille

**Affiliations:** 1 Douglas Mental Health University Institute, Montreal, QC Canada; 2 Department of Neurosciences, McGill University, Montreal, QC Canada; 3 Department of Psychiatry, McGill University, Montreal, QC Canada; 4 Department of Psychology, McGill University, Montreal, QC Canada

**Keywords:** Cognitive neuroscience, Learning and memory, Electrophysiology

## Abstract

The learning ability of individuals within the schizophrenia spectrum is crucial for their psychosocial rehabilitation. When selecting a treatment, it is thus essential to consider the impact of medications on practice effects, an important type of learning ability. To achieve this end goal, a pre-treatment test has to be developed and tested in healthy participants first. This is the aim of the current work, which takes advantage of the schizotypal traits present in these participants to preliminary assess the test’s validity for use among patients. In this study, 47 healthy participants completed the Schizotypal Personality Questionnaire (SPQ) and performed a semantic categorization task twice, with a 1.5-hour gap between sessions. Practice was found to reduce reaction times (RTs) in both low- and high-SPQ scorers. Additionally, practice decreased the amplitudes of the N400 event-related brain potentials elicited by semantically matching words in low SPQ scorers only, which shows the sensitivity of the task to schizotypy. Across the two sessions, both RTs and N400 amplitudes had good test–retest reliability. This task could thus be a valuable tool. Ongoing studies are currently evaluating the impact of fully deceptive placebos and of real antipsychotic medications on these practice effects. This round of research should subsequently assist psychiatrists in making informed decisions about selecting the most suitable medication for the psychosocial rehabilitation of a patient.

## Introduction

Recent literature and clinical practice have consistently shown that patients with schizophrenia spectrum disorders encounter significant challenges when learning new tasks and adapting to novel social environments^1–3^. These learning deficits have a huge negative impact on their vocational and psychosocial rehabilitation outcomes^4–6^. Dopaminergic dysfunction is one of several mechanisms that could underlie these deficits. This dysfunction seems to exist both in patients with schizophrenia spectrum disorders and in subclinical people with high schizotypal traits^7–9^. Dysregulated dopamine levels may induce an aberrant assignment of salience to irrelevant internal or external stimuli, leading to an "overlearning" of neutral or irrelevant information. Dysregulated dopamine may also be associated with difficulties in learning reward-predictive information^10–13^. Irrespective of the specific neurochemical mechanisms at play, these difficulties impede the rehabilitation of patients. Therefore, a test that rapidly quantifies the learning improvements induced by medication in a particular patient may prove instrumental in selecting the best antipsychotic for his/her rehabilitation.

Schizotypy is increasingly recognized as a psychological construct within the schizophrenia spectrum. It is a set of personality traits, such as unusual thinking patterns and difficulties in interpersonal communication, which are associated with a propensity to develop full-blown schizophrenia^14^. Several studies suggest that schizotypy and schizophrenia not only overlap at the phenomenological level of symptoms but also have significant genetic, cognitive, and neurobiological similarities^15^. For example, factor analysis-based studies have shown that the positive, negative, and disorganized dimensions of schizophrenia can also be found in non-clinical schizotypy^16–18^. Individuals with high schizotypy also exhibit similarities in neurocognitive performance with patients diagnosed with schizophrenia^19^. Moreover, antipsychotic medications have comparable effects on neurocognition in both groups^15^. Assessing the degree of schizotypy within the general population to determine each participant’s position on the normality-to-schizophrenia continuum thus offers valuable insights into understanding the risk, development, expression, trajectory, and treatment of the full-blown disorder^20–23^. This assessment can be done using the Schizotypal Personality Questionnaire (SPQ), which is based on the DSM-III-R criteria for the diagnosis of schizophrenia and is also used in schizophrenia patients^24–26^. From the perspective of antipsychotic drug development, schizotypy research has many advantages, such as the availability of reliable and objective psychometric self-questionnaires^27^ and the absence of confounding effects of disease chronicity and previous exposure to antipsychotic medications^28^.

Investigating the effects of practice in a laboratory setting using experimental psychology methods may be valuable for better specifying and understanding the cognitive changes and learning deficits observed within the spectrum of schizophrenia. Effects of practice, which refer to the *spontaneous* learning that naturally occurs between two testing sessions, reflect various cognitive abilities, such as procedural memory and the development of test-taking strategies. Such abilities appear to be essential for optimizing performance in many daily activities^29–31^. In healthy participants, practice can effectively remedy poor cognitive performance. Such natural practice effects differ from what is often called learning potential (LP)^5^, which involves improvements induced by training interventions that occur between the test and retest sessions (i.e., test-train-test approaches)^32,33^. In a meta-analysis^34^, scores in executive function and attention tasks were found to improve over time in healthy subjects, while no significant improvement was found in individuals within the schizophrenia spectrum. Another meta-analysis^35^ also reported that during the course of the disorder, such patients did not improve in performance across repetitions of cognitive tests compared to the control group. Similar abnormalities in practice effects have been found in subclinical people with schizotypy, such as poorer performance at the retest session than at the test session in tasks such as continuous performance^36^, Wisconsin card sorting, and verbal fluency tests^37^. This lack of practice effects reflects an abnormal learning of new skills within the schizophrenia spectrum. As mentioned by the American Academy of Clinical Neuropsychology (AACN), “There is an obvious need for more data on normal change trajectories for all types of measures with all types of demographic variables and patient groups^38^”.

On the other hand, it remains unclear whether antipsychotic medications have an *acute* impact on practice effects. The effects of practice mentioned above were observed between testing sessions that were often separated by several days, weeks, or even months. However, today, we know that antipsychotic medications can improve certain clinical symptoms of patients much faster. For instance, Agid et al.^39^ found a significant dose-related effect (20 mg vs. 2 mg) on the early psychosis factor and scores of a positive symptom subscale 4 hours after intramuscular (IM) injection of ziprasidone. Patients with schizophrenia taking 10 mg of olanzapine IM showed overall relief on the psychosis factor of the Brief Psychiatric Rating Scale (BPRS) 2 hours after^40^. Furthermore, even only one hour after the first injection, a reduction in the Excited Component of the Positive and Negative Syndrome Scale (PANSS-EC) scores was evident in the group having 10 mg olanzapine IM^41–44^ and in the group having the oral disintegrating tablet^45^. In fact, such early clinical effects are not surprising. Previous studies have reported that a single dose of 400–450 mg quetiapine gives rise to transiently high (58–64%) striatal dopamine D2 occupancy 2 to 3 hours after its intake^46^. Nevertheless, the speed at which an intake of antipsychotics acts on cognitive deficits has not been measured yet. Given the pivotal role that learning ability plays in the social rehabilitation of patients, it could be useful to investigate practice effects within a short time frame first, such as 90 minutes. The choice of duration is close to the maximum plasma concentration reached after the intake of one pill for most antipsychotics^47^ and short enough to be usable in clinical practice. Together with a rapid decrease in clinical symptoms,  acute cognitive improvements might predict the efficacy of the medication for the psychosocial rehabilitation of a patient in the long run.

Bizarre and aberrant semantic processing is one of the central features of schizophrenia. It can be assessed by semantic priming paradigms, such as lexical decision tasks (LDTs), which are used in schizophrenia spectrum research. In these tasks, participants are presented with strings of letters (e.g., toble) and are required to decide whether or not each string is a real English word. Semantics are manipulated by presenting a related prime word (e.g., chair) or an unrelated one (e.g., car) before each target word (e.g., table)^48–51^. A variety of studies have confirmed a semantic priming effect. The time taken to make the lexical decision, or reaction time (RT), is shorter for target words preceded by such related words^52^. In people with schizophrenia attributes (SzAs, i.e., schizophrenia patients and subclinical people with schizotypal traits), this RT priming is smaller than in healthy controls with low schizotypal traits when the time between the onset of the priming word and that of the string of letter is longer than 500 ms^53^. On the other hand, the N400 event-related brain potential (ERP) has also been found to depend on semantic priming. This ERP has a negative-going electrical polarity and a maximum voltage of around 400 ms after the onset of the stimulus^54–56^. Like RTs, N400 amplitudes are smaller for target words preceded by a related word than for target words preceded by an unrelated word, which is usually interpreted as indexing easier processing of semantic information^54^. Researchers have investigated N400 priming impairments in people with SzAs and showed that their N400 amplitudes in response to unprimed targets were generally a bit smaller, while in response to primed targets, they were somewhat larger than those of healthy controls, resulting in reduced N400 effects^57–61^. N400 semantic priming deficits have been shown to predict worse symptomatic and functional outcomes after one^62^ and two years^63^. While abnormalities in other electrophysiological indexes, such as the mismatch negativity, P3a, and auditory steady-state response, have been observed in schizophrenia patients^64^, their applicability to study practice effects is limited because they only reflect automatic pre-attentive functions.

Both RTs and N400 amplitudes have already been used to measure practice effects. In healthy participants, significant effects of practice and priming on both measures were found over a 3-month test-to-retest delay^51^. Interestingly, at least two other studies also report different effects of practice on the effects of priming. According to the results of Besche-Richard et al.^48^, practice does not change priming effects in healthy participants. In schizophrenia patients, the behavioral semantic priming remained impaired, whereas their smaller N400 priming effect and their clinical symptoms were found to be significantly improved at their one-year retest session. However, Kiang et al.^50^ reported that in healthy participants, the amplitude of the N400 semantic priming effect decreased by about 1.22 µV from the test to the retest session spaced one week apart. A similar, albeit non-significant (likely due to a small sample size), effect of practice on priming effects was also found on RTs.

To address some of the discrepancies across the studies mentioned above, a particular semantic categorization task^65^ was chosen to measure practice effects on RTs and N400 amplitudes for the present study. In this task, the question-word "ANIMAL?" is systematically presented at the beginning of each trial, reminding of the task instruction. It is followed by an exemplar (e.g., dog) or a non-exemplar word (e.g., table) of the animal semantic category. Participants have to decide whether or not the target word belongs to this category. This task was first chosen because it uses language stimuli, which are the types of stimuli patients frequently encounter when interacting with others or when receiving instructions at the workplace. It was also chosen because it focuses directly on the meaning of the stimuli rather than on judging whether or not a string of letters is a real word. Indeed, it is the understanding of meanings that is of critical importance for rehabilitation. Focusing on such semantics also yields more robust N400 effects^66^, which enhances the reproducibility of results. In addition, like LDTs, this semantic task enables the recording of both the behavioral responses and brain activity, which allows for the identification of the stages of processing that undergo changes and those that do not change with practice in a patient. This task was also chosen because its difficulty is moderate, with error rates smaller than 10% in healthy participants. Using such low-difficulty level tasks prevents uncertainty about response accuracy and reduces the potential for disengagement and/or shifts in cognitive strategies used during the task. Finally, presenting an instruction word with each trial refreshes participants' working memory (WM) and effectively circumvents WM deficiencies observed in people with SzAs^67,68^.

In summary, our end goal is to explore the potential utility of practice effect measures, an important type of learning ability, within a short timeframe by using a particular semantic categorization task to detect and quantify the rapid effects of medication on these measures. Indeed, these rapid effects could potentially predict the therapeutic efficacy of an antipsychotic on the psychosocial rehabilitation of a patient with schizophrenia in the long term. To achieve this end goal, it is necessary to first evaluate these practice effects and the reliability of these measures in subclinical individuals according to their schizotypal traits. This is the aim of the present work. To ensure that the test remains short enough for easy use with patients in clinical settings, a short time interval (i.e., 90 minutes) was used between the first session (referred to as the study session) and the second session (referred to as the test session).

## Results

### Questionnaires

The SPQ scores of our participants covered a relatively wide range of the continuum between low and high schizotypy, namely, from 0 to 38 (out of 74, the maximal score). The mean of the total SPQ score of all participants was 17.4 (*SD* = 11.0). High- and low-schizotypy subgroups did not significantly differ in terms of sex, age, and level of education (see Table 1). There was a SPQ x session interaction on the level of anxiety (F (1, 45) = 10.4, *p* = 0.002, *ηp*^2^ = 0.19). It was further explored by pairwise comparisons, which revealed that the mean anxiety level of the high SPQ subgroup (mean = 28.4, SD = 17.3) was significantly higher than that of the low SPQ subgroup (mean = 15.0, SD = 13.9) before the start of the experiment. STAI scores of the two groups significantly increased along with the experiment. This increase was larger for the low- than for the high-SPQ subgroup, so there was no significant difference between the mean anxiety level of the low SPQ subgroup (mean = 59.4, SD = 4.5) and that of the high SPQ subgroup (mean = 56.6, SD = 5.7) after the experiment. On the contrary, fatigue did not significantly increase during the experiment. Fatigue of the high SPQ subgroup (mean = 38.2, SD = 17.0) was significantly higher than that of the low SPQ group (mean = 21.9, SD = 17.6), both before and after the experiment (F (1, 45) = 11.2, *p* = 0.002, *ηp*^2^ = 0.20).

**Table 1:** Demographic characteristics of participants.

	Low schizotypy	High schizotypy
	(*N* = 23)	(*N* = 24)
Sex: male, % (*N*)	43.5% (10.0)	45.8% (11.0)
Age, *M (SD, RANGE)*	23.7 (3.1, 19–30)	22.5 (3.0, 18–30)
Years of Education, *M (SD, RANGE)*	15.4 (1.6, 13–18)	14.8 (1.7, 11–18)
Total SPQ, *M (SD, RANGE)*	7.5 (4.9, 0–15)	26.9 (5.2, 18–38)
Anxiety (pre-experiment), *(SD, RANGE)*	15.0 (14.0, 0–50)	28.4 (17.3, 0–64)
Anxiety (post-experiment), *(SD, RANGE)*	59.4 (4.5, 52–67)	56.6 (5.7, 48–67)
Fatigue (pre-experiment), *(SD, RANGE)*	20.4 (17.0, 0–63)	40.8 (16.0, 0–75)
Fatigue (post-experiment), *M (SD, RANGE)*	23.4 (18.4, 0–69)	36.6 (18.1, 6–75)

### Practice effects in the behavioral data and N400 amplitudes

#### Behavioral data

**Figure 1:**
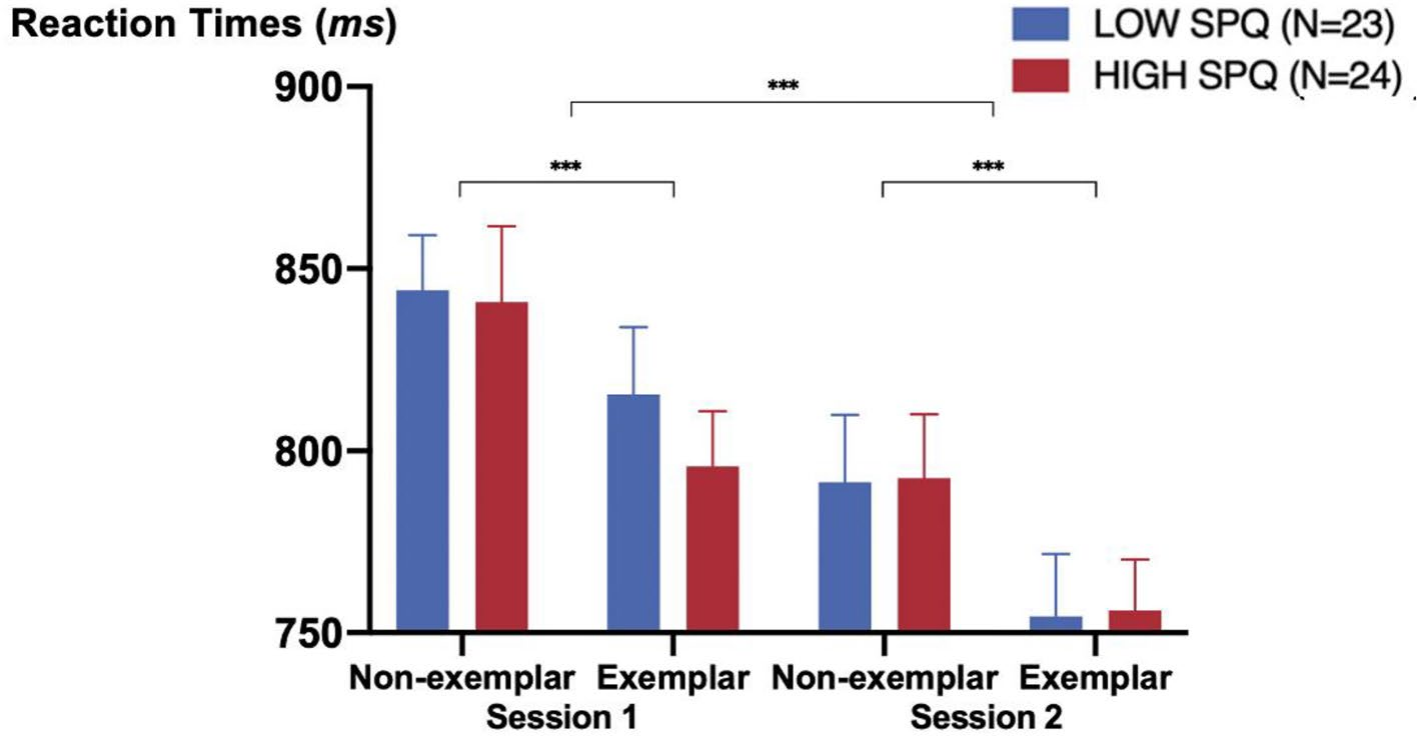
Mean reaction times on non-exemplar and exemplar conditions in session 1 and session 2 (N = 47). Error bars display the standard error. *** are for* p *< 0.001.

The mean of response accuracy (RA) was 93.0% (SD = 7.3, range = 54–100). Participants' RAs in the non-exemplar condition (mean = 95.8%, SD = 4.1) were significantly higher than in the exemplar condition (mean = 90.1%, SD = 8.6) (F (1, 45) = 32.1, *p* = 9.72 × 10^–7^, *ηp*^2^ = 0.42). The debriefing session of prior studies^69^ showed that those few errors in the exemplar condition were largely attributed to insects, which were not considered to be animals by some participants. Participants' RTs in session 2 (mean = 774 ms, SD = 82) were significantly faster than in session 1 (mean = 824 ms, SD = 86) (F (1, 45) = 37.7, *p* = 1.92 × 10^–7^, *ηp*^2^ = 0.46). RTs of the non-exemplar condition (mean = 817 ms, SD = 90) were significantly slower than in the exemplar condition (mean = 780 ms, SD = 81) (F (1, 45) = 39.3, *p* = 1.25 × 10^–7^, *ηp*^2^ = 0.47). There was no category x session interaction on RTs (Fig. 1).

#### N400 amplitudes

**Figure 2:**
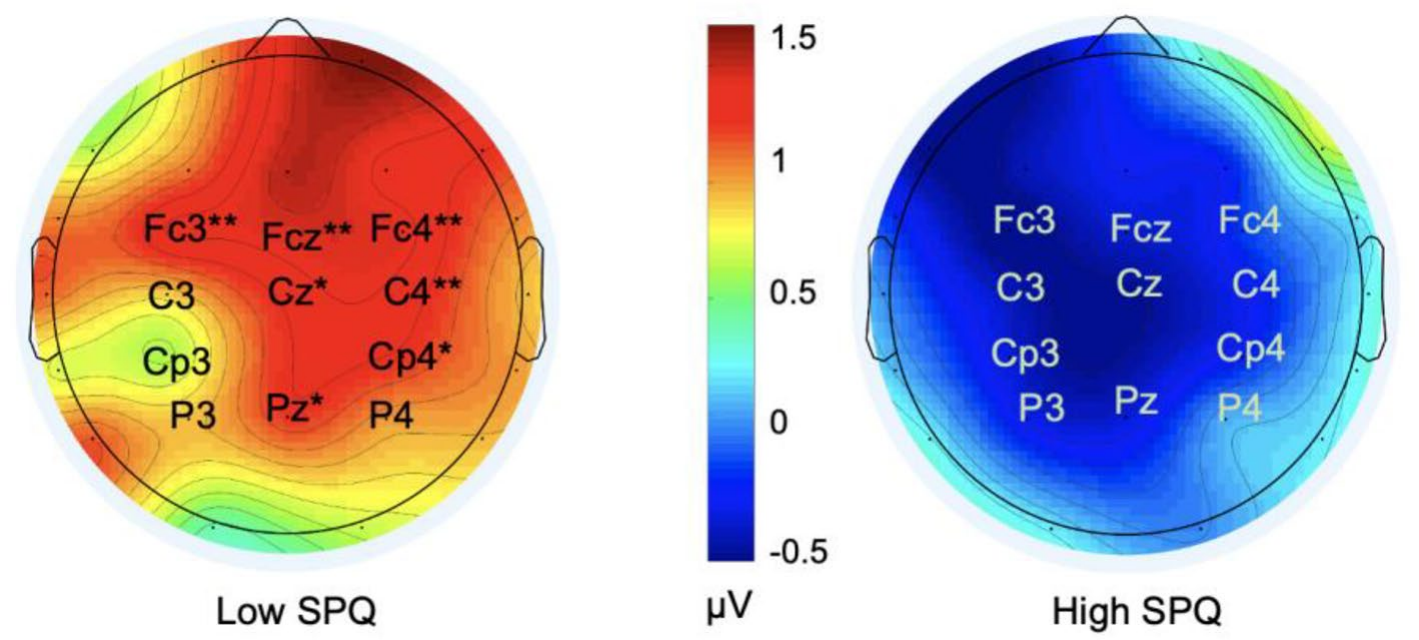
Spline interpolated isovoltage scalp maps illustrating (1) the effect of practice on the N400 amplitudes of the exemplar condition in participants with low SPQ scores (N = 23) and (2) the absence of such an effect in the high SPQ (N = 24) subgroup. The values coded by the map colors correspond to the results of the subtraction of the mean ERP voltages of session 1 from those of session 2 in the N400 time window (300 ms – 500 ms). * are for 0.05 > *p *> 0.01. ** are for 0.01 > *p *> 0.001.

The omnibus ANOVA performed on N400 mean amplitudes revealed several statistically significant interactions: session x SPQ (F (1, 45) = 5.3, *p* = 0.026, *ηp*^2^ = 0.1), electrode x category (F (10, 450) = 3.6, *p* = 0.002, *ηp*^2^ = 0.1), and session x category (F (1, 45) = 4.1, *p* = 0.05, *ηp*^2^ = 0.08). We then focused on each category condition to identify the source of the session x SPQ x electrode interaction. There was a SPQ x session interaction in the exemplar condition (F (1, 45) = 6.4, *p* = 0.015, *ηp*^2^ = 0.12). Post-hoc ANOVAs revealed that N400 amplitudes in session 2 (− 0.3 µV) were significantly smaller than in session 1 (− 1.5 µV) only in the exemplar condition and only for participants with low SPQ scores (Fig. 2). This was not the case for participants in the high SPQ subgroup.

**Figure 3:**
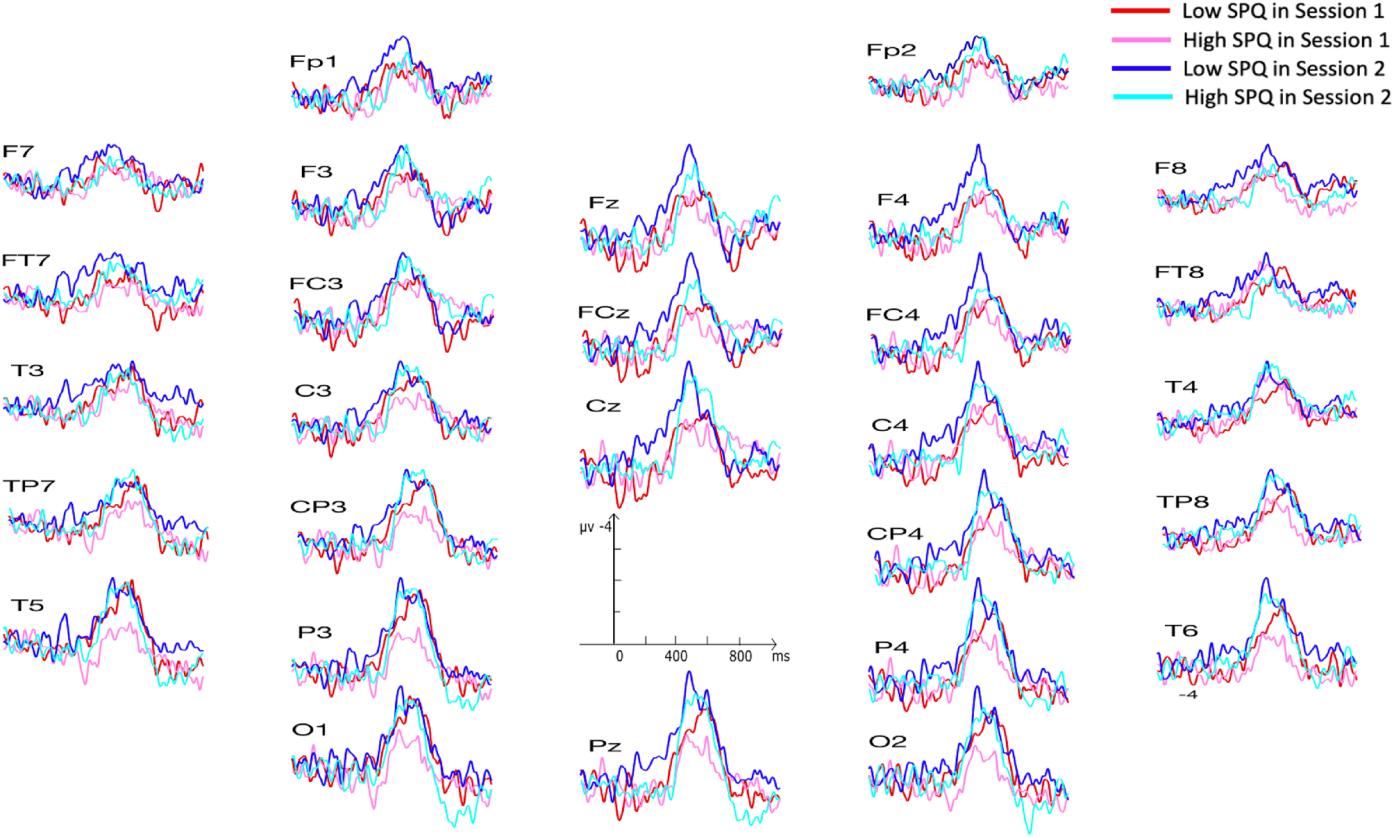
Illustrating the larger N400 effects in session 2 than in session 1. N400 effects were obtained by subtracting the ERPs of the exemplar condition from the ERPs of the non-exemplar condition. Those of session 1 are the dark red lines for the low SPQ subgroup (N = 23) and the light red lines for the high SPQ subgroup (N = 24). Those of session 2 are the dark blue lines for the low SPQ subgroup and the light blue lines for the high SPQ subgroup.

There was a marginally significant effect of session on the N400 effect, (F (1, 45) = 4.1, *p* = 0.05, *ηp*^2^ = 0.08). The N400 effect of session 2 (− 1.6 µV) was a bit larger than that of session 1 (− 1.0 µV) (see Fig. 3). There was neither an effect of SPQ on the N400 effect nor any interaction including this factor. ERP figures for raw N400 amplitudes for the exemplar and non-exemplar conditions are provided in the Supplementary Materials.

### Test–retest and internal consistency reliability of behavioral data and N400 amplitudes

#### Behavioral data

**Figure 4:**
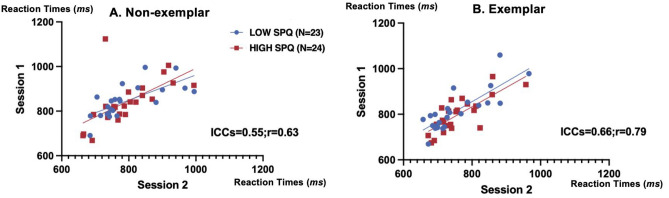
Illustrating the reliability of behavioral data across the two sessions by plotting the mean RTs of session 1 as the x coordinate of each disk/square-participant and the mean RTs of session 2 as its y coordinate (N = 47).

To test whether behavioral data obtained in this particular semantic task were reliable, we examined whether participants who had, for instance, faster mean reaction times at session 1 relative to other participants also had faster mean reaction times at session 2 relative to other participants. As illustrated in Fig. 4, these correlations were significantly positive for the non-exemplar condition (Pearson's r = 0.63, *p* = 9.1 × 10^–7^, 95% confidence interval (CI) [0.38, 0.86]; intraclass correlation coefficients (ICCs) = 0.55, *p* = 7.0 × 10^–7^, 95% CI [0.19, 0.75]); and for the exemplar condition (Pearson's r = 0.79, *p* = 2.0 × 10^–11^, 95% CI [0.67, 0.88]; ICCs = 0.66, *p* = 1.6 × 10^–11^, 95% CI [0.088, 0.86]). No significant reliability was found in the STAI-Y questionnaire, whereas there was a high test–retest reliability of the fatigue questionnaire (Pearson's r = 0.84, *p* = 2.6 × 10^–13^, 95% CI [0.70, 0.92]; ICCs = 0.84, *p* = 1.4 × 10^–13^, 95% CI [0.73, 0.91]).

**Figure 5:**
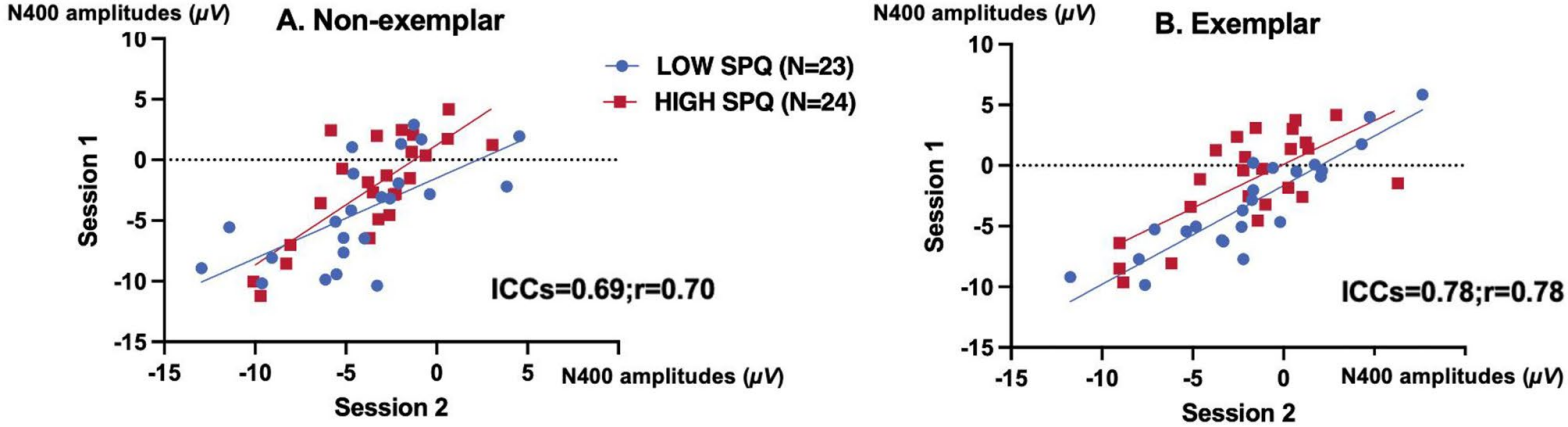
Illustrating the reliability of N400 amplitudes at Cz across the two sessions by plotting the N400 mean voltages of session 1 as the x coordinate of each circle-participant together with N400 of session 2, the y coordinate of each circle-participant (N = 47).

The reliability of N400 amplitudes was assessed at Cz and for a central cluster of 11 electrodes (Fc3/4, C3/4, Cp3/4, P3/4, Pz, Cz, and Fcz), where N400 effects obtained with written word stimuli are usually found to be maximal. We also examined whether participants had similar scores from different subsets of trials (i.e., internal consistency reliability). Table 2 displays the internal consistency reliability (ICR) and the test–retest reliability (TRR) found. In general, the mean N400 amplitudes of both non-exemplar and exemplar conditions had good reliability. The mean reliability of N400 amplitudes of the exemplar condition was better than that of the non-exemplar condition at Cz and the central cluster. Figure 5 shows scatterplots of correlations between session 1 and session 2 for N400 amplitudes at Cz.

**Table 2:** Internal consistency reliability (ICR) and test–retest reliability (TRR) of N400 mean amplitudes for non-exemplar and exemplar conditions in the two sessions at Cz and central cluster. CI is 95% confidence interval. Central cluster includes: Fc3/4, C3/4, Cp3/4, P3/4, Pz, Cz and Fcz.

	Mean amplitude							
	Non-exemplar				Exemplar			
	ICR	CI	TRR	CI	ICR	CI	TRR	CI
Cz	0.88	[0.84, 0.92]	0.69	[0.51, 0.82]	0.88	[0.82, 0.92]	0.80	[0.67, 0.89]
Central Cluster	0.87	[0.82, 0.91]	0.55	[0.31, 0.73]	0.86	[0.81, 0.91]	0.73	[0.55, 0.85]

### Correlations between N400 amplitudes and SPQ

**Figure 6:**
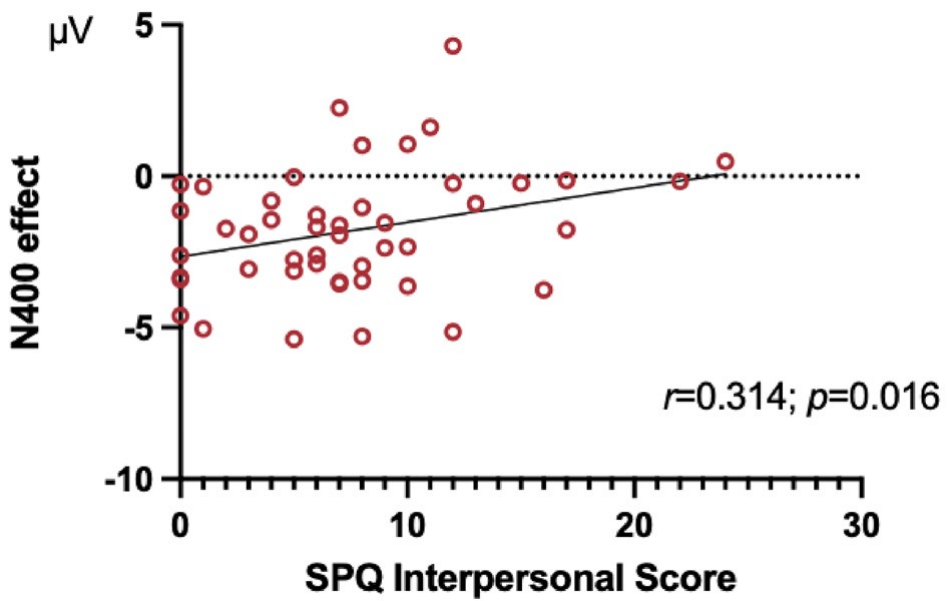
Illustrating the correlation between SPQ Interpersonal scores and N400 effects of session 2 at Pz by plotting SPQ Interpersonal scores as the x coordinate of each circle-participant together with N400 effects of session 2 at Pz, the y coordinate of each circle-participant (N = 47).

We did not find a strong correlation between SPQ scores and N400 amplitudes (most Pearson’s r were smaller than 0.3). Only one significant correlation was found between SPQ Interpersonal scores and N400 effects of session 2 at Pz (Pearson's r = 0.31, *p* = 0.016) (Fig. 6), possibly because the highest SPQ score in our sample was not very high compared to those seen in schizophrenia patients^24^.

## Discussion

The present study is an initial step towards evaluating a task that could enable a rapid selection of the most effective antipsychotic medication to improve the practice effect of a patient. Indeed, these improvements have the potential to facilitate his/her rehabilitation and subsequently ameliorate his/her psychosocial outcomes. We conducted a study involving 47 healthy individuals who were tested twice in a particular semantic categorization task, taking into account their schizotypal traits. Practice effects were observed over the course of 90 minutes separating the two sessions of this task, indicated by a significant reduction in RTs from session 1 to session 2. Secondly, good test–retest reliability was observed, and the test was found to be sensitive to the mild-to-moderate schizotypal traits of our subclinical participants.

A high response accuracy was observed, indicating that participants were attending to the stimuli and performing the task. In addition, as mentioned, practice effects were observed. Participants responded 50 ms faster in session 2 than in session 1, consistent with the results found in three previous studies: in Kiang et al.^50^, who reported a 30 ms decrease over a one-week interval; in Besche-Richard et al.^48^, who found a 48 ms decrease over a one-year interval, and in Yu et al.^51^, who found a 45 ms RT reduction in a lexical decision task at the retest session 3 months later.

RTs and N400 measures had good test–retest reliability, which is consistent with previous studies^49–51^. The test–retest reliability for the N400 at Cz was 0.69 for the non-exemplar and 0.80 for the exemplar conditions, and the internal consistency reliability was 0.88 for the two conditions. In other studies, the reliability of N400 amplitudes at Cz has been reported as 0.69 for unrelated and 0.74 for related targets within a one-week interval^50^; 0.75 for unrelated and 0.55 for related targets within a three-month interval^51^. The high test–retest reliability of N400 amplitudes found in healthy controls in the present study is comparable to, or better than, other putative ERP biomarkers, like the amplitudes of P300 and that of the mismatch negativity^70,71^. The present results also further support the N400 as a potential bioindicator in longitudinal treatment studies of the schizophrenia spectrum. However, we did not find retest reliability on the N400 priming effect. One possible reason is the relatively small proportion of related prime-target pairs (RP) employed in this study, at approximately 33.3%. Notably, Stolz et al.^72^ reported that RP and reliability are inversely proportional.

The N400 semantic priming effect of session 2 was 0.5 µV larger than that of session 1. This effect of practice on the N400 semantic priming effect did not interact with the SPQ group. This finding is inconsistent with previous studies. For example, Besche-Richard et al.^48^ reported that, in healthy participants, practice does not change semantic priming effects at their one-year retest session. Kiang et al.^50^ found that the N400 semantic priming effect decreased by about 1.22 µV from the test to the retest session split one week apart. One possible explanation for these discrepancies is that in the present study, the interval between the two sessions was 90 minutes, which is much shorter than in other studies, that is, one year and one week, respectively. Barnett et al.^73^ proposed that the size of the practice effect is inversely proportional to the interval between the test and retest sessions.

Secondly, we found that N400 amplitudes for the exemplar condition decreased significantly with practice in participants with low SPQ scores, but not in participants with high SPQ scores. Interestingly, this difference between SPQ subgroups was not reflected in the RTs, suggesting that N400s may index processes other than those indexed by RTs. As a matter of fact, in tasks like the present one, where participants had to provide a response as fast as possible (in addition to being as accurate as they could), RTs mainly depend on *activations*, as repeatedly found when testing the so-called race models of RTs^74^. These models account for RTs obtained, for instance, in divided attention tasks, when two target stimuli are presented simultaneously. There, participants respond faster than when only one stimulus is presented. According to race models, the two target stimuli are processed independently. RTs are then determined by the stimulus that triggers or activates the response first: the winner of the race. This could also be the case here, as our word stimuli (e.g., dog) activate more than one meaning corresponding to a response (e.g., the meaning of pet and the meaning of mammal). The meaning that is processed the fastest will be the one activating the response first. In contrast to RTs, N400s might index *inhibitory* mechanisms^75–77^. The decrease of N400 amplitudes observed with practice in participants with low SPQ could be a consequence of a decrease in the activation of inappropriate representations by the question-word "ANIMAL?". Less inhibition would then be necessary during the processing of exemplar target words, hence, the reduced N400 amplitudes would be found in these conditions. If this were the case, it would mean that one of the effects of practice is the development of more focused processing and that such improvements are compromised by a higher degree of schizotypal traits. It would also mean that a notable inhibition remains to be performed at the occurrence of the target word in participants with higher SPQ scores.

Besche‐Richard et al.'s results^48^ can be used to further support a difference between RTs and N400s. At a retest session one year later, they found that symptom-relieved schizophrenia patients still had deficits in priming effects on RTs, whereas their priming effects on N400 significantly improved. These findings also suggest that the effect of practice on N400s might be a better predictor of clinical improvement than the effects of practice on RTs. This claim can be reinforced by the overall slower RTs usually exhibited by patients with schizophrenia, responsible for an inflation of the relative differences in RTs between non-exemplar and exemplar conditions, which can sometimes lead to spurious behavioral results^78^.

One potential limitation of this study could be the repetition effect. Indeed, the task included identical stimuli across the two consecutive sessions. The observed practice effects could thus be partly attributed to repetition effects. This repetition is very well-known to cause a robust decrease in the amplitudes of the raw N400 and of the N400 effects that are obtained in lexical decisions^79–81^. Surprisingly, such a robust N400 decrease was neither observed for non-exemplar words nor for both types of words in the high SPQ group. What was observed here was a moderate N400 decrease only for exemplar words and only in the low SPQ subgroup. This absence of a robust and systematic effect of repetition on the N400 could be due to the other task that participants had to perform between the semantic task of session 1 and the semantic task of session 2. This task used words other than those used in the semantic task. It also pertained to the meaning of these words, but in a very different way. This intervening task could have prevented the classical robust and systematic effects of repetition on the N400s.

Classically, the repetition of stimuli also induces a decrease in RTs^56,82,83^. The faster RTs observed in session 2 than in session 1 could thus be due to such effects. Nevertheless, this possibility is unlikely because the three previous aforementioned studies reported RT reductions of a similar magnitude to the one reported here, despite employing much longer intervals between the two sessions. Repetition effects on RTs tend to be more pronounced with shorter delays^84–86^. Consequently, if the observed RT decrease was solely attributable to stimulus repetition, it would have been substantially larger than the 50.4 ms that we reported. Instead, it is more plausible that this RT decrease is primarily a result of the pure practice effects associated with the task itself. This claim is further supported by the enduring nature of procedural memory, which remains almost unchanged over the years^87,88^. This could account for the fact that the sizes of the RT reductions observed in our study are similar in magnitude to those in the three previous studies.

The level of participants’ anxiety should be considered when interpreting the results. The high SPQ subgroup exhibited significantly higher anxiety levels compared to the low SPQ subgroup before the experiment. This is consistent with previous research showing that the SPQ score is positively correlated with trait anxiety^89^. Participants’ anxiety could have thus contributed to the lack of practice effect in the N400 amplitudes among high SPQ scorers in the exemplar condition. Indeed, this high level of pre-experiment anxiety may partially impede flexibility and the ability to maintain and develop focus during practice^90^. A meta-analysis of 177 studies has shown that self-reported measures of anxiety are reliably associated with poorer performance on measures of working memory capacity^91^. Anxiety-related behavioral phenotypes seem to be related to disrupted prefrontal cortex neural activity in healthy individuals^92,93^. Anxiety levels of both groups increased during the experiment. Some other electroencephalography (EEG) studies also reported increased levels of anxiety at the end of the experiment^94,95^. This may be due to the novelty of the experience, concerns about the expected outcome, the discomfort of wearing electrode caps, the need to control blinks and eye movements, etc.

Next, a normative sample of schizophrenia patients should be tested longitudinally to know whether the initial change of practice effects induced by the chosen medication predicts the long-term (e.g., a year) functional outcome of a patient. In addition, the study of such effects may be critical not only in clinical trials but also in all studies using placebo controls. In fact, improvements in patients receiving placebos are likely to be at least partly due to practice effects and not only to the expectation bias inherent to placebo-controlled designs^31^. Future studies should thus compare practice effects in patients receiving a placebo to those of individuals who are not receiving anything. Other studies investigating the impact of antipsychotic medications (e.g., olanzapine and risperidone) on practice effects could then control for the placebo effect of the studied medication. This would be a more rigorous way to see whether the impact of medication on practice effects predicts patients' psychosocial rehabilitation, and whether such effects can thus be used to select what would be considered the best medication for a particular patient.

## Methodology

### Participants

Were selected among candidates who answered our English or French online advertisements in a variety of social media (e.g., Kijiji, Facebook, and the McGill Classified Ads website). Participants had to be native English or French speakers with at least ten years of education in either language. The sample size was calculated by power analysis using G*Power 3.1. We referred to previous studies where the effect size of the practice effect on RTs was around 0.33^51^. Calculations showed that a minimum of 22 participants per group was necessary to achieve 80% power. To ensure replicability, we used 47 participants (45 Anglophones and 2 Francophones). All participants were right-handed and had no previous history of a neurological condition, no medical condition that compromises brain function, and no head injury with a loss of consciousness longer than 5 minutes. We also excluded those with a personal history of DSM-IV Axis I psychiatric disorder, a family history of schizophrenia or bipolar disorder, alcohol or substance abuse disorder, or current use of a psychotropic medication. 47 healthy participants between the ages of 18 and 30 (*mean* = 23.1, *SD* = 3.1, 21 females) were retained. All participants provided written informed consent form prior to participation. This study was approved by the Douglas Ethics Review Board (project number: IUSMD-06-42). All research was performed in accordance with relevant guidelines/regulations and the Declaration of Helsinki.

*Psychometric scales* Before the EEG recording session, participants also completed a set of questionnaires in their preferred language (English or French). This set included the State-Trait Anxiety Inventory Form Y (STAI-Y State)^96^ and a self-created questionnaire evaluating fatigue (see Supplementary Materials). STAI-Y has high-reliability coefficients of 0.92 for the State and 0.90 for the Trait scales (Form Y), respectively^97^. The questionnaire set also included the Schizotypal Personality Questionnaire (SPQ), which has high internal reliability (coefficient alpha > 0.90) and test–retest reliability (r = 0.82)^98^. This questionnaire was initially designed to measure the severity of schizotypal personality traits in the general population. It has been widely used as a psychometric tool in research^99^. Nevertheless, it is based on the DSM-III-R criteria used to diagnose schizophrenia and is also used with schizophrenia patients^24–26^. The STAI-Y and fatigue questionnaires had to be completed twice, that is, once before and once after the experiment, in order to evaluate the effect of sessions on fatigue and anxiety as potential confounding factors. In contrast, SPQ was administered only once before the EEG session, as SPQ total scores remained relatively stable over time^100^.

### Procedure

Upon arrival, participants completed a demographics questionnaire, where they provided information regarding their sex, age, and level of education. Immediately after completing the set of questionnaires, the EEG recording session began, during which participants provided responses in the semantic categorization task (session 1) for about 15 minutes. Right after, for approximately 15 minutes, participants had to perform another task using word stimuli and focusing on their meaning. However, that task was peripheral to the objectives of the present study and is therefore not discussed. A one-hour lunch break was then given to all participants. It was followed by session 2, where the target words of the semantic task remained the same as those used in session 1.

### Stimuli

The semantic task was identical to the one used in Debruille et al.^69^, which has both an English and a French version. Participants completed tasks in their preferred language, English or French. It included 180 trials, each made of two serially presented words. In two-thirds of the trials, the first word was the question-word "ANIMAL?", followed by an exemplar (e.g., dog) or a non-exemplar (e.g., table) of the animal category. Participants had to decide if the target word belonged to the animal category as accurately and rapidly as possible by pressing a "Yes" for matching targets or a "No" key for mismatching targets with their right index finger. Exemplar and non-exemplar words were matched for the number of letters and frequency of usage using the Content et al.^101^ database for the French words and the Kucera et al.^102^ counts for the English words. In one-third of the trials, the first word was "INACTION", which meant that participants should not respond to the second stimulus of the trial.

**Figure 7:**
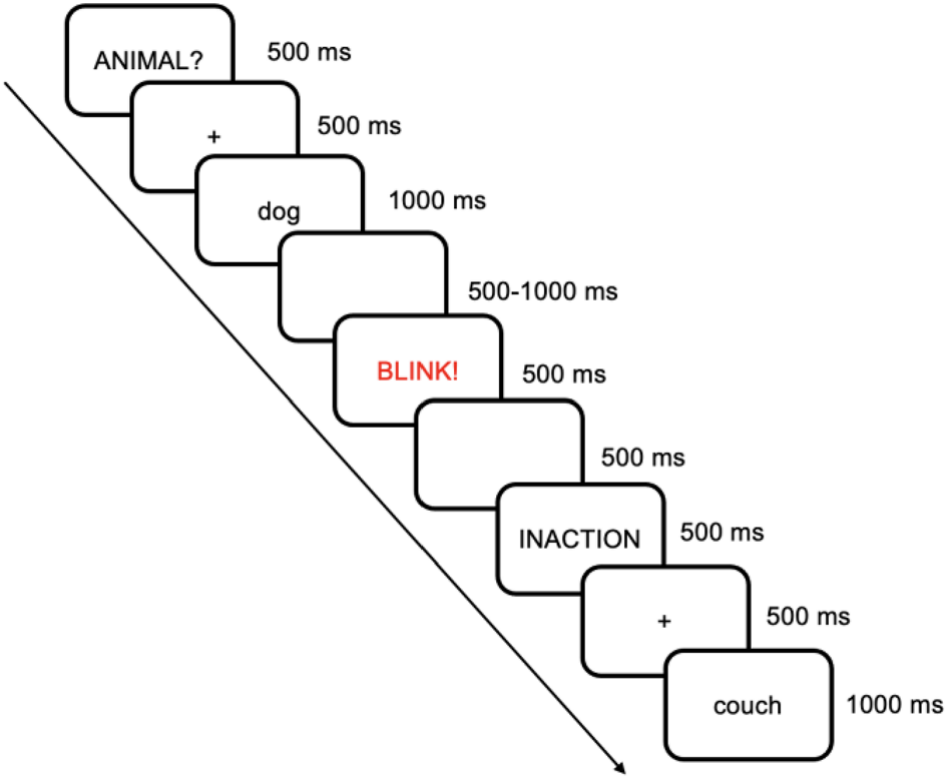
The procedure of the semantic categorization task.

The priming word "ANIMAL?" (or "INACTION") appeared in the center of the screen for 500 ms and was replaced by a fixation cross for 500 ms. This cross was followed by the target word for 1000 ms or until a valid keypress occurred (Fig. 7). Such a long stimulus onset asynchrony (SOA), that is, such a long time between the onset of the prime and the onset of the target word, was chosen because it allows observing robust N400 priming deficits in schizophrenia patients^103–105^. Moreover, long SOAs have been proven to boost test–retest reliability^72,106^. Each target word was then replaced, 1.5–2 s later, by the word “Blink”, which lasted for 500 ms.

### Data acquisition

The time taken to make the exemplar/non-exemplar decision (i.e., the RT) was recorded at each trial. The EEG was recorded from 28 tin electrodes mounted on an Electro-Cap International (ECI) cap. The following sites of the international 10–20 system were used: Fp1/2, F3/4, Fc3/4, C3/4, Cp3/4, P3/4, O1/2, Fz, Fcz, Cz, Pz, F7/8, Ft7/8, T3/4, Tp7/8, and T5/6. The right earlobe was used as the reference, and the ground was placed 2 cm anterior to Fz. The impedance was measured before the experiment using a 30 Hz current and was kept below 5 KΩ. An electronic notch filter was used to reduce the 50 Hz EM noise coming from power lines. The high- and low-pass filters had their half amplitude cut-off set at 0.01 Hz and 100 Hz, respectively. EEG signals were digitized at a 248 Hz sampling rate.

### Data processing and measures

The data were processed in MATLAB using the EEGLAB toolbox with the ERPLAB extension. An independent component analysis (ICA) was first used to identify and remove artifactual components, such as blinks, eye movements, and myograms. The infomax algorithm ICA was performed on a copy of the continuous EEG that was high-pass filtered at 1 Hz and low-pass filtered at 30 Hz. The resulting ICA weight matrix and sphering matrix were then applied to the continuous 0.1–30 Hz filtered EEG by using the ICLabel EEGLAB extension to signal artifactual independent components (ICs) that had > 20% of chance of being muscle activity or > 8% of chance of being eye activity^107^. These ICs were then systematically subtracted from this continuous 0.1–30 Hz filtered EEG, as in Finke et al.^108^, Goregliad et al.^109^, and Markey et al.^110^.

Only trials including behavioral responses performed between 300 and 2500 ms post-onset were retained. This was done to eliminate trials where participants did not pay enough attention, were too hesitant, or, on the contrary, provided responses that were too prompt, revealing an absence of real stimulus evaluation. The EEG epochs of those trials were taken from 200 ms pre-stimulus to 1000 ms post-stimulus. Their baselines were set by computing the mean voltages in the − 200 to 0 ms time window for each electrode and by subtracting this mean value from each point of the − 200 to 1000 ms epoch. Trials with voltages exceeding the amplitude range of −/+100 μV for the 4 frontal electrodes (Fp1/2, F7/8) and of − /+75 μV for the remaining 24 electrodes were rejected, as well as epochs containing flat lines persisting for more than 100 ms. Participants were included in the analysis only if a minimum of 30 trials in each condition survived these artifact rejection criteria. Although the ERP Reliability Analysis (ERA) Toolbox identifies the minimum number of trials needed to be 16 in the non-exemplar condition and 15 in the exemplar condition for adequate reliability, we were not only interested in grand average ERPs but also in individual participant studies using Monte Carlo methods. For this purpose, we kept a cut-off of 30 trials (50% of all trials) for each condition. Tables 3 and 4 provide the average number of accepted trials and the standardized measurement error (SME) for the N400 amplitudes on each condition across participants. An ERP was computed by averaging all the remaining EEG epochs corresponding to one condition (e.g., exemplar target words). The measures of N400 amplitudes used were the mean voltages of the signal of each electrode in the 300–500 ms time window. The N400 priming effect was computed by subtracting the ERPs elicited by exemplars (i.e., names of animals) from the ERPs elicited by non-exemplars (i.e., names of objects).

**Table 3:** Average numbers of accepted trials, N400 mean amplitudes at Cz/central cluster and SME (standardized measurement error) by condition across participants in session 1. Central cluster includes: Fc3/4, C3/4, Cp3/4, P3/4, Pz, Cz and Fcz.

		Session 1			
		Low SPQ		High SPQ	
		Non-exemplar	Exemplar	Non-exemplar	Exemplar
Numbers of trials accepted	M (SD)	48.0 (6.2)	44.7 (7.4)	46.6 (7.5)	43.5 (11.8)
	range	34.0–56.0	25.0–56.0	32.0–57.0	18.0–59.0
N400 at Cz (µV)	M (SD)	− 2.2(4.3)	− 3.1 (4.1)	− 2.2 (4.2)	− 1.3 (4.0)
	range	− 10.4–2.9	− 9.9–5.8	− 11.2–4.2	− 9.6–4.2
N400 at central cluster (µV)	M (SD)	− 2.6 (3.4)	− 1.5 (3.2)	− 1.3 (3.3)	− 0.5 (3.1)
	range	− 10.4–4.6	− 9.9–5.9	− 11.2–5.8	− 9.6–7.9
SME	M (SD)	1.0 (0.2)	1.0 (0.2)	1.1 (0.3)	1.2 (0.4)
	range	0.7–1.3	0.7–1.7	0.3–1.7	0.3–2.5

**Table 4:** Average numbers of accepted trials, N400 mean amplitudes at Cz/central cluster and SME (standardized measurement error) by condition across participants in session 2. Central cluster includes: Fc3/4, C3/4, Cp3/4, P3/4, Pz, Cz and Fcz.

		Session 2			
		Low SPQ		High SPQ	
		Non-exemplar	Exemplar	Non-exemplar	Exemplar
Numbers of Trials accepted	M (SD)	49.9 (6.9)	44.3 (9.0)	49.2 (6.1)	46.0 (7.5)
	range	31.0–57.0	30.0–58.0	31.0–57.0	29.0–57.0
N400 at Cz (µV)	M (SD)	− 4.2 (4.2)	− 1.8 (4.5)	− 3.5 (3.3)	− 1.9 (3.8)
	range	− 13.0–4.6	− 11.8–7.7	− 10.1–3.1	− 9.0–6.3
N400 at central cluster (µV)	M (SD)	− 2.2 (3.0)	− 0.3 (3.2)	− 2.2 (2.8)	− 0.9 (3.3)
	range	− 13.0–4.6	− 11.8–7.7	− 10.5–5.5	− 11.2–7.6
SME	M (SD)	0.9 (0.2)	0.9 (0.2)	1.0 (0.3)	1.1 (0.3)
	range	0.5–1.3	0.5–1.3	0.4–1.5	0.3–1.7

### Statistical analyses

In the current study, the total SPQ score was used to median split participants into high- and low-schizotypy subgroups. Questionnaire scores, mean reaction times (RTs), and mean accuracies (RAs) were analyzed with mixed-model repeated-measures analysis of variance (ANOVA) using a multivariate approach. SPQ subgroup was the between-subjects factor, whereas category (animal vs. object names) and session (Session 1 vs. Session 2) were the within-subject factors.

Mean N400 amplitudes and N400 effects (subtracting the ERP elicited by exemplars from the ERP evoked by unrelated non-exemplars) were also analyzed with mixed-model repeated-measures ANOVA having the same between- and within-subject factors, to which electrode was added as a within-subject factor (11 levels: Fc3/4, C3/4, Cp3/4, P3/4, Pz, Cz, and Fcz). As previous literature has shown that the N400 effect is usually most prominent at central and parietal sites^54^, we also computed a mean of all these electrodes. These simpler statistics are provided in the Supplementary Materials.

For the reliability analysis, test–retest and internal consistency reliability of N400 amplitudes for non-exemplar and exemplar conditions between session 1 and session 2 were processed in the ERP Reliability Analysis (ERA) Toolbox^111^. The acceptable reliability cut-off was set as 0.7 due to the novelty of assessing the reliability of this particular semantic task^111^. For the test–retest reliability of behavioral data and questionnaires, intraclass correlation coefficients (ICCs) and Pearson’s r correlation coefficients between session 1 and session 2 were calculated. A “Two-Way mixed effects model” and “absolute agreement” were used in the ICCs calculation^51,112^.

All statistical analysis were performed with IBM SPSS Statistics (version 27). The Greenhouse and Geisser’s adjustment of the degrees of freedom was used to compensate for the heterogeneity of variances across electrodes. In those cases, the original F-values and degrees of freedom are provided together with the corrected p-values. The Benjamini–Hochberg false discovery rate (B-H FDR) procedure was then used to evaluate the significance of each p-value of each series of tests. P-values were thus first ranked from the most to the least significant. One B-H FDR threshold for each of these p-values was then computed by dividing its rank by the total number of tests and by multiplying the result by the false discovery rate chosen (i.e., 10%). The p-value was declared significant if it was smaller than that threshold.

### Supplementary Information

Supplementary Information.

## Data Availability

All analysis scripts and datasets (behavioral and EEG) can be obtained from the corresponding author upon request.
