# Supplementary material for: Quantifying the effects of practicing a semantic task according to subclinical schizotypy
Source: Sci Rep. 2024 Feb 5;14:2900. doi: 10.1038/s41598-024-53468-4 (PMC10844607; doi:10.1038/s41598-024-53468-4)
Supplement: Supplementary file 1 — Supplementary Information. [file 41598_2024_53468_MOESM1_ESM.docx]

**Supplementary materials**

# Table of contents

# [Fatigue and anxiety questionnaires](#Fatigue)

# 1.1 [Participant's instructions for the first fatigue questionnaire](#P1)

# 1.2 [Table 1. First fatigue questionnaire](#table1)

# 1.3 [Participant's instructions for the second fatigue questionnaire](#P3)

# 1.4 [Table 2. Second fatigue questionnaire](#table2)

# 1.5 [Participant's instructions for the anxiety questionnaire](#P5)

# 1.6 [Table 3. Anxiety questionnaire](#table3)

# [Results of statistical analysis of mean voltage of the 11 electrodes of the ROI](#ROI)

# [Grand average ERPs of the exemplar and non-exemplar target words](#ERP)

# 3.1 [Figure 1. Grand average ERPs of session 1 (top) and session 2 (bottom) for the Low SPQ group](#F1)

# 3.2 [Figure 2. Grand average ERPs of session 1 (top) and session 2 (bottom) for the High SPQ group](#F2)

# 3.3 [Figure 3. Grand average ERPs of session 1 (top) and session 2 (bottom)](#F3)

# [Figure 4. Grand average ERPs of non-exemplar (top) and exemplar (bottom) condition](#F4)

# [List of the stimuli used](#S)

# Fatigue and anxiety questionnaires

Over the entire course of the study, two fatigue and anxiety questionnaires were used in succession to measure the energy and anxiety levels of the participants. In order to unify the analysis, we selected, from both questionnaires, the questions that reflected the current energy and anxiety levels of the participants and standardized them using the percentage maximum possible (POMP) method (Cohen et al., 1999). POMP scores are calculated by POMP = 100*(raw - min)/(max - min) with raw = original mean score of variables (items) with valid values, min = minimum possible value, and max = maximum possible value.

## Participant's instructions for the first fatigue questionnaire:

Please answer each question on a scale of 1 to 10 to indicate how you currently feel - that is, in the present moment. Answer ALL items even if unsure of your answer. When you have finished, check over each one to make sure you have answered them all (1-Lowest; 10-Extremely high).

## Table 1. First fatigue questionnaire

|  | **1** | **2** | **3** | **4** | **5** | **6** | **7** | **8** | **9** | **10** |
| --- | --- | --- | --- | --- | --- | --- | --- | --- | --- | --- |
| How is your level of energy? | **○** | **○** | **○** | **○** | **○** | **○** | **○** | **○** | **○** | **○** |
| How sleepy are you? | **○** | **○** | **○** | **○** | **○** | **○** | **○** | **○** | **○** | **○** |

## *Questions selected from the first fatigue questionnaire and used for standardization.*

## Participant's instructions for the second fatigue questionnaire:

The following ten statements refer to how you currently feel. Please select the answer to each question that is applicable to you. Please give an answer to each question (1-Never; 5-Always).

## Table 2. Second fatigue questionnaire

|  | **1** | **2** | **3** | **4** | **5** |
| --- | --- | --- | --- | --- | --- |
| I am bothered by fatigue; | **○** | **○** | **○** | **○** | **○** |
| Physically, I feel exhausted | **○** | **○** | **○** | **○** | **○** |
| I feel no desire to do anything | **○** | **○** | **○** | **○** | **○** |
| Mentally, I feel exhausted | **○** | **○** | **○** | **○** | **○** |

## *Questions selected from second fatigue questionnaire and used for standardization.*

## Participant's instructions for the anxiety questionnaire:

A number of statements which people have used to describe themselves are given below. Read each statement and then select the appropriate number next to the statement to indicate how you feel right now, that is, at this moment. There are no right or wrong answers. Do not spend too much time on any one statement but give the answer which seems to describe your present feelings best (1-No at all; 4-Very much so).

## Table 3. Anxiety questionnaire

|  | **1** | **2** | **3** | **4** |
| --- | --- | --- | --- | --- |
| I feel calm | **○** | **○** | **○** | **○** |
| I feel secure | **○** | **○** | **○** | **○** |
| I am tense | **○** | **○** | **○** | **○** |
| I feel at ease | **○** | **○** | **○** | **○** |
| I feel upset | **○** | **○** | **○** | **○** |
| I am presently worrying over possible misfortunes | **○** | **○** | **○** | **○** |
| I feel comfortable | **○** | **○** | **○** | **○** |
| I feel self-confident | **○** | **○** | **○** | **○** |
| I feel nervous | **○** | **○** | **○** | **○** |
| I am jittery | **○** | **○** | **○** | **○** |
| I am relaxed | **○** | **○** | **○** | **○** |
| I feel content | **○** | **○** | **○** | **○** |
| I am worried | **○** | **○** | **○** | **○** |
| I feel pleasant | **○** | **○** | **○** | **○** |

## *Questions selected from anxiety questionnaire and used for standardization.*

Cohen, P., Cohen, J., Aiken, L. S., & West, S. G. (1999). The problem of units and the circumstance for POMP. *Multivariate behavioral research,* 34(3), 315-346. <https://doi.org/10.1207/s15327906mbr3403_2>

# Results of the statistical analysis of the mean voltage at 11 electrodes in the region of interest

Raw N400 amplitude: The omnibus ANOVA performed on N400 mean amplitudes revealed several statistically significant interactions: session x SPQ (F (1, 45) = 5.3, *p* = 0.026, *ηp2* = 0.1) and session x category (F (1, 45) = 4.1, *p* = 0.05, ηp2 = 0.08). The post-hoc ANOVAs run there revealed that N400 amplitudes in session 2 (-0.3 µV) were significantly smaller than those of session 1 (-1.5 µV) only in the exemplar condition and only for participants with low SPQ scores. This was not the case for participants in the high SPQ subgroup.

N400 effect: There was a marginally significant effect of session on the N400 effect, (F (1, 45) = 4.1, *p* = 0.05, *ηp2* = 0.08). The N400 effect of session 2 (-1.6 µV) was larger than that of session 1 (-1.0 µV). There was neither an effect of SPQ on the N400 effect nor any interaction including this factor.

# Grand average ERPs of the exemplar and non-exemplar target words

*
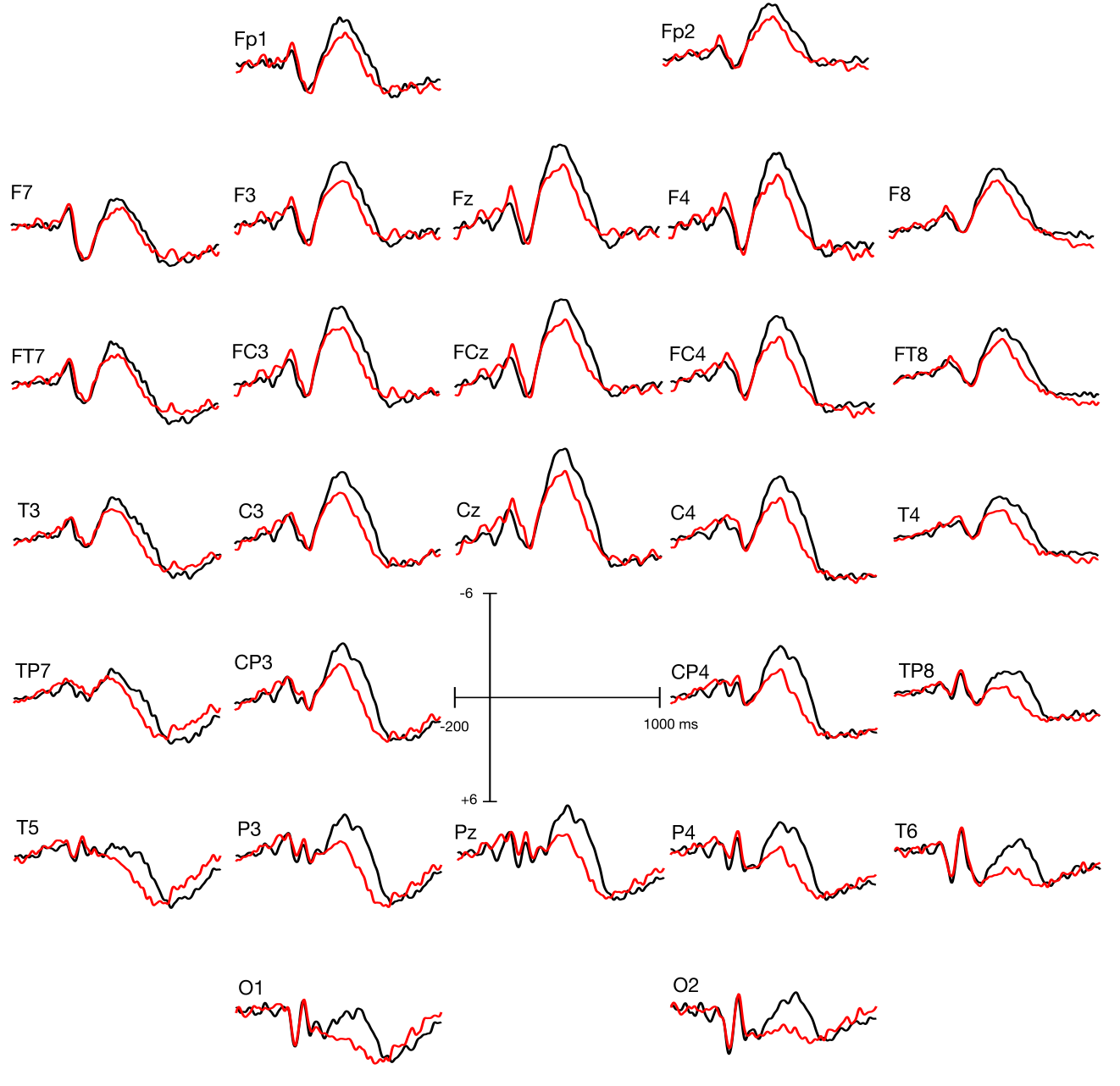
*

*
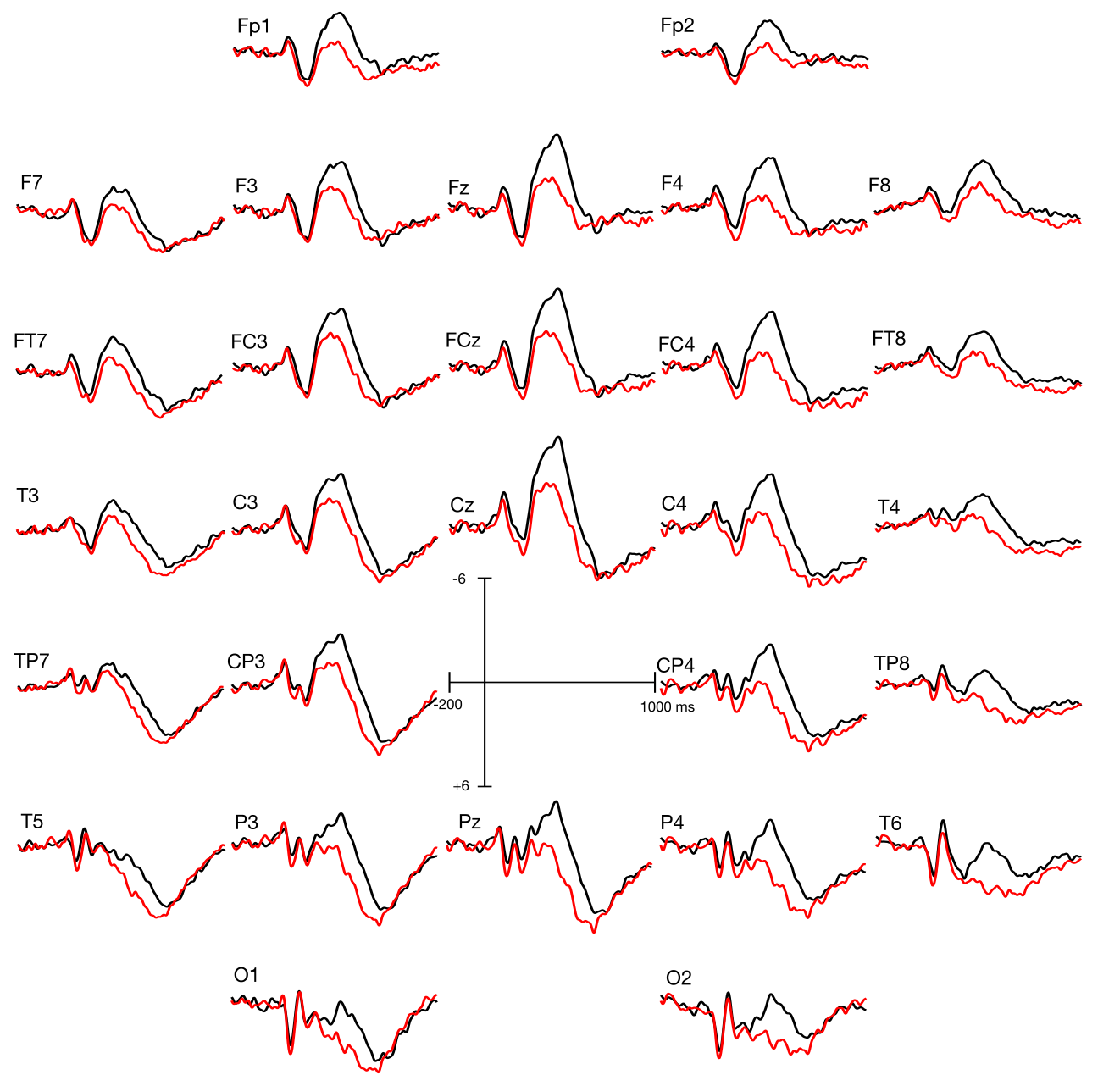
*

*Figure 1. Grand average ERPs of session 1 (top) and session 2 (bottom) for the participants of the low SPQ subgroup****.*** *Black lines correspond to the non-exemplar condition, red lines correspond to the exemplar condition (N=23).*

*
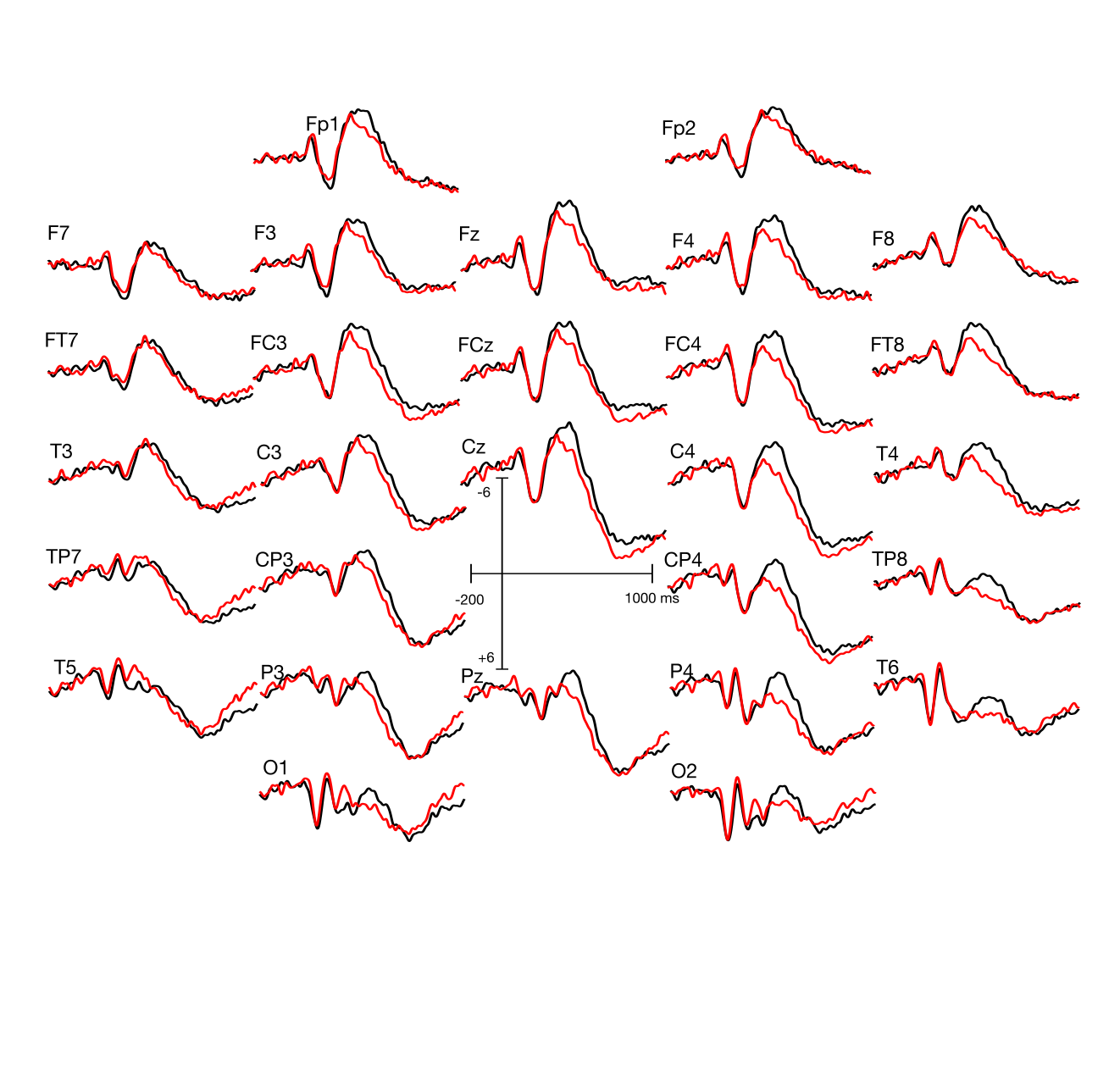

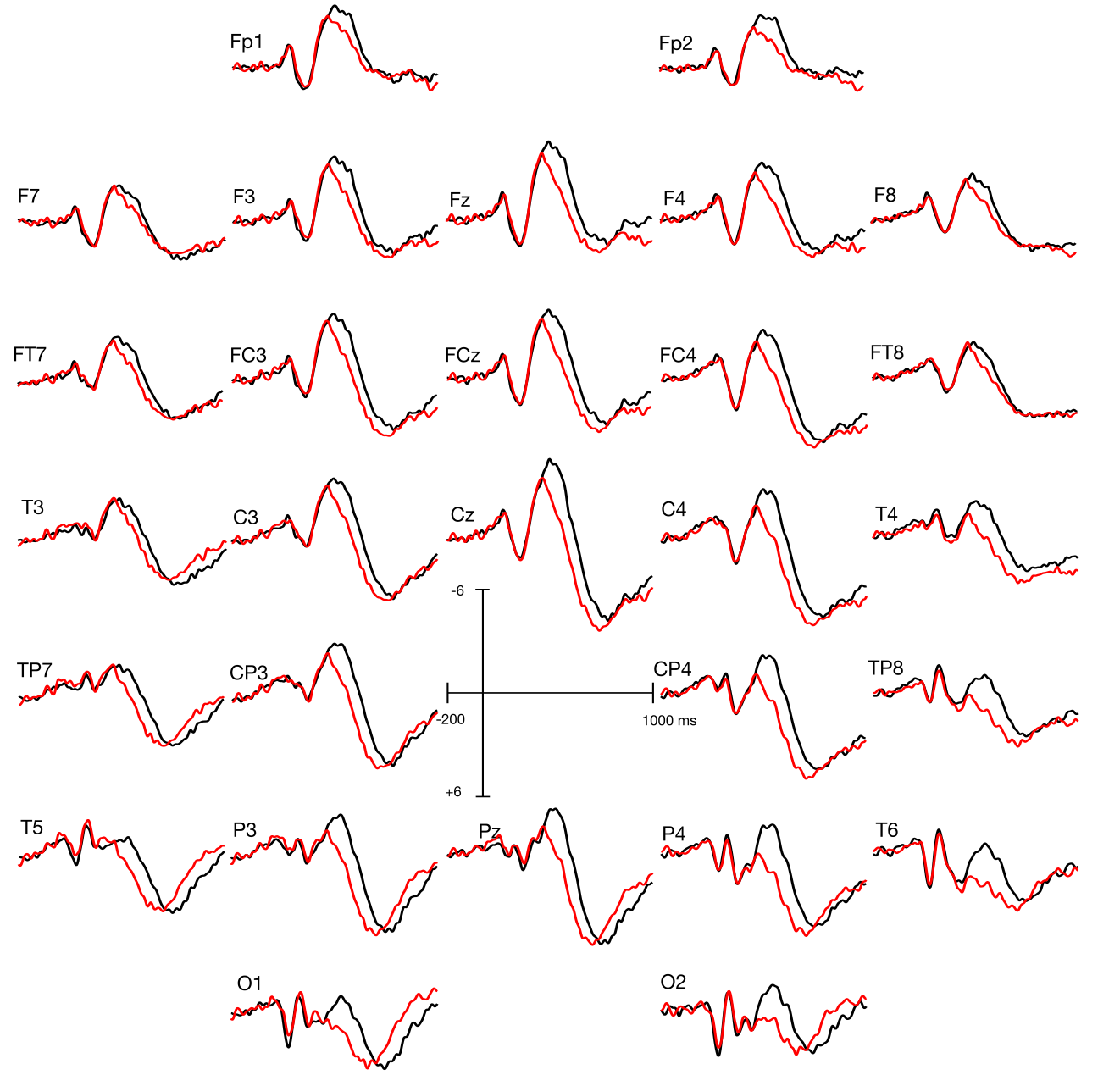
*

*Figure 2. Grand average ERPs of session 1 (top) and session 2 (bottom) for the high SPQ subgroup. Black lines correspond to the non-exemplar condition, red lines correspond to the exemplar condition (N=24).*

*
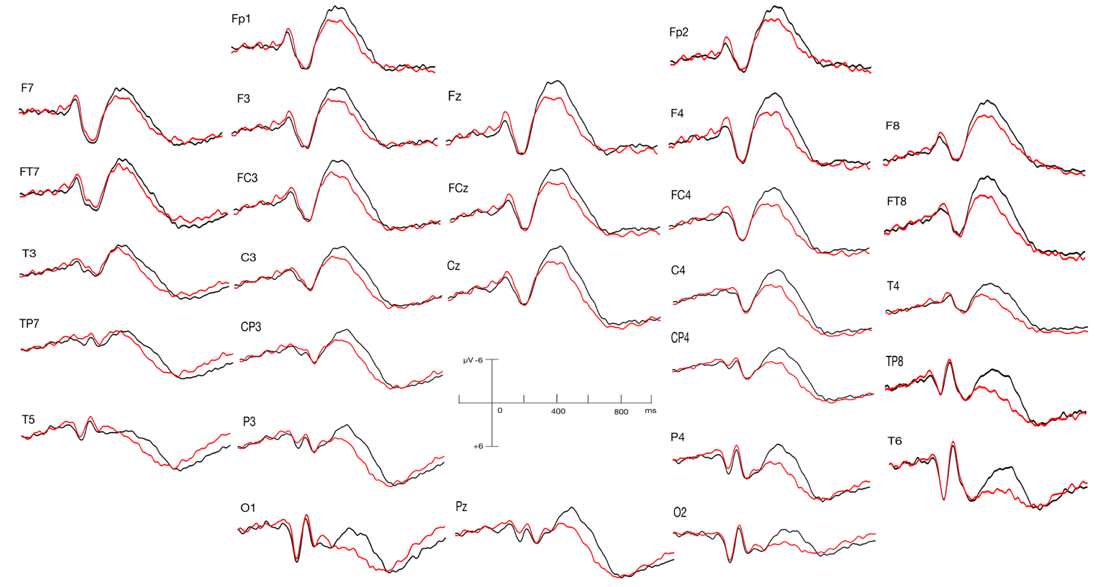
*

*
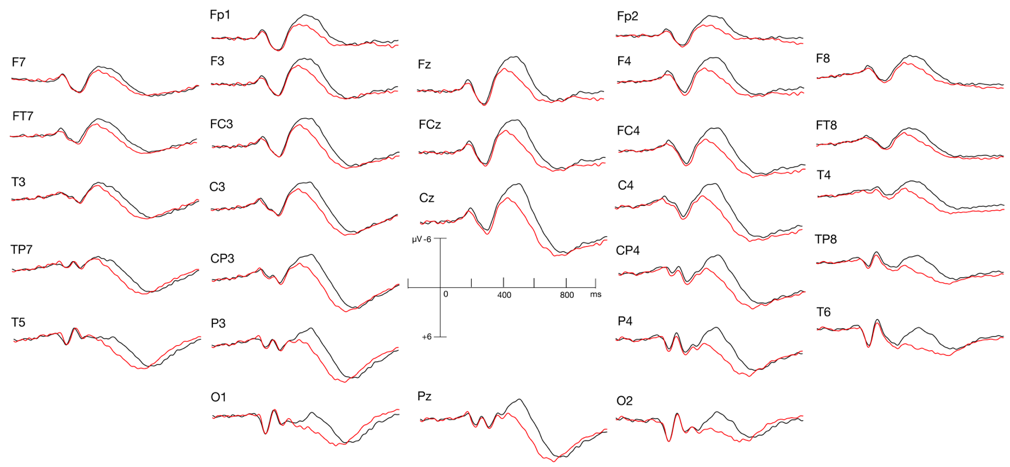
*

*Figure 3. Grand average ERPs of session 1 (top) and session 2 (bottom). Black lines correspond to the non-exemplar condition, red lines correspond to the exemplar condition (N=47).*

*
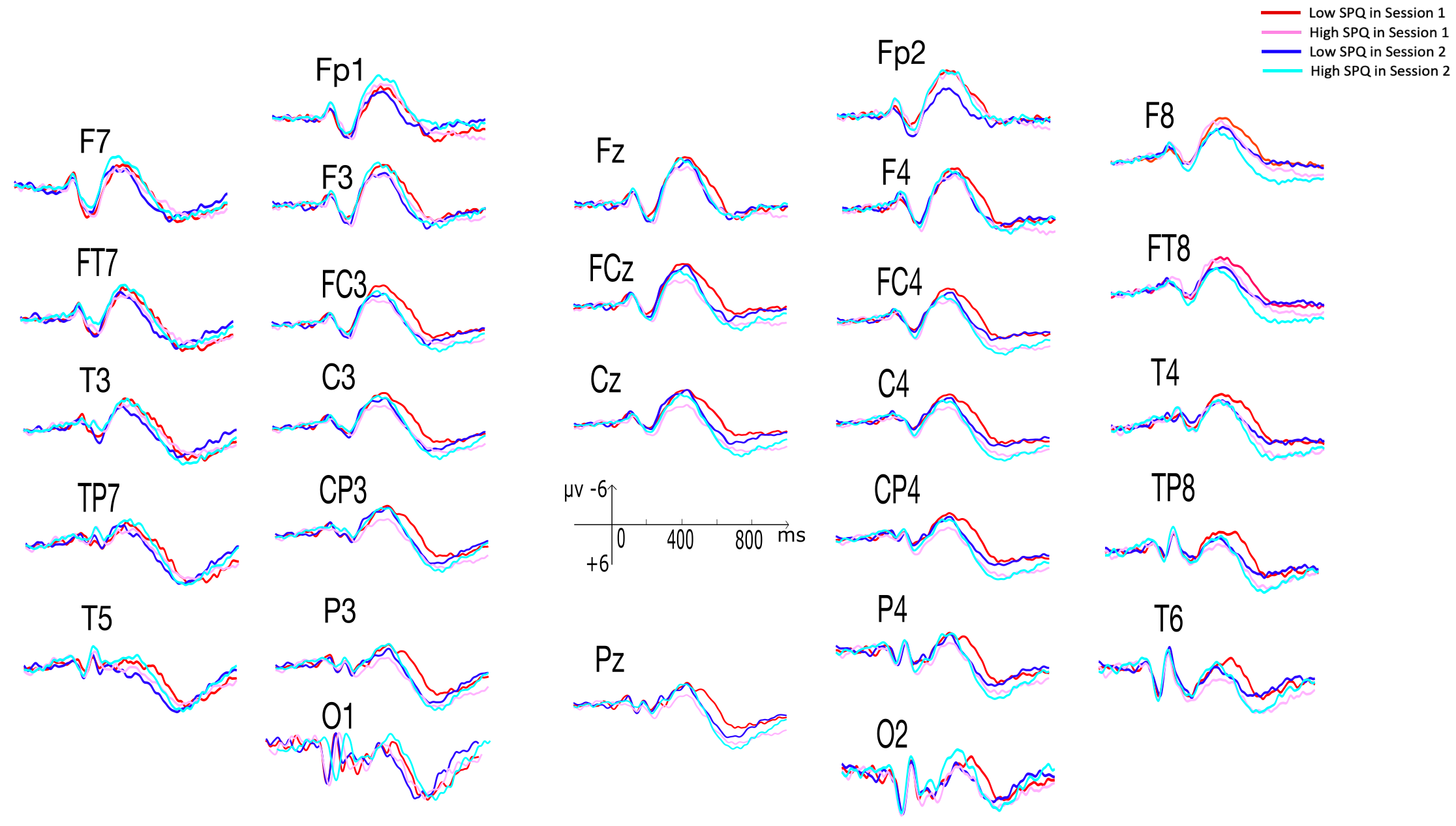
*

*
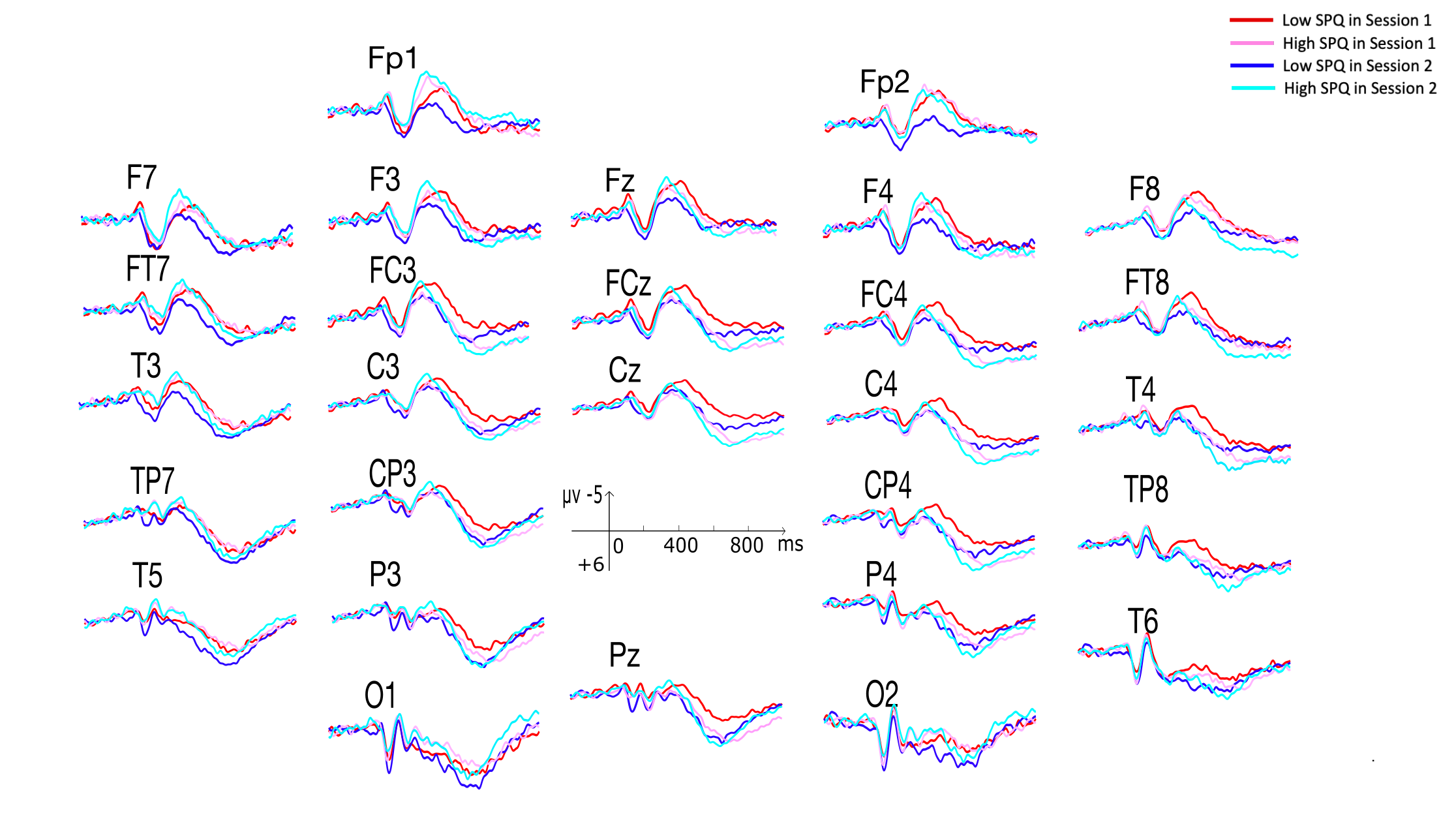
*

*Figure 4. Grand average ERPs of non-exemplar (top) and exemplar (bottom) conditions. Those of session 1 are the dark red lines for the low SPQ subgroup (N = 23) and the light red lines for the high SPQ subgroup (N = 24). Those of session 2 are the dark blue lines for the low SPQ subgroup and the light blue lines for the high SPQ subgroup.*

# List of the stimuli used

| INACTION | closet |
| --- | --- |
|  |  |
| ANIMAL? | basin |
|  |  |
| INACTION | shirt |
|  |  |
| ANIMAL? | alligator |
|  |  |
| ANIMAL? | puma |
|  |  |
| ANIMAL? | panther |
|  |  |
| ANIMAL? | cloth |
|  |  |
| INACTION | parachute |
|  |  |
| ANIMAL? | octopus |
|  |  |
| INACTION | fleece |
|  |  |
| ANIMAL? | alarm |
|  |  |
| INACTION | bus |
|  |  |
| ANIMAL? | rabbit |
|  |  |
| INACTION | leopard |
|  |  |
| ANIMAL? | swallow |
|  |  |
| INACTION | deer |
|  |  |
| ANIMAL? | whale |
|  |  |
| ANIMAL? | lace |
|  |  |
| ANIMAL? | viper |
|  |  |
| ANIMAL? | lizard |
|  |  |
| ANIMAL? | pigeon |
|  |  |
| ANIMAL? | wasp |
|  |  |
| ANIMAL? | keyboard |
|  |  |
| ANIMAL? | helmet |
|  |  |
| INACTION | cylinder |
|  |  |
| INACTION | bicycle |
|  |  |
| ANIMAL? | spoon |
|  |  |
| ANIMAL? | bill |
|  |  |
| INACTION | ski |
|  |  |
| ANIMAL? | camel |
|  |  |
| ANIMAL? | earthworm |
|  |  |
| ANIMAL? | satchel |
|  |  |
| ANIMAL? | match |
|  |  |
| ANIMAL? | dummy |
|  |  |
| INACTION | flea |
|  |  |
| ANIMAL? | boa |
|  |  |
| INACTION | hedge |
|  |  |
| ANIMAL? | cod |
|  |  |
| ANIMAL? | boot |
|  |  |
| ANIMAL? | parrot |
|  |  |
| INACTION | icicle |
|  |  |
| INACTION | jelly-fish |
|  |  |
| ANIMAL? | eagle |
|  |  |
| ANIMAL? | ambulance |
|  |  |
| INACTION | pants |
|  |  |
| ANIMAL? | heron |
|  |  |
| ANIMAL? | lamb |
|  |  |
| INACTION | panda |
|  |  |
| ANIMAL? | husky |
|  |  |
| INACTION | piranha |
|  |  |
| ANIMAL? | directory |
|  |  |
| ANIMAL? | cap |
|  |  |
| ANIMAL? | monkey |
|  |  |
| ANIMAL? | flask |
|  |  |
| INACTION | duck |
|  |  |
| ANIMAL? | rake |
|  |  |
| ANIMAL? | reptile |
|  |  |
| ANIMAL? | fish |
|  |  |
| ANIMAL? | stork |
|  |  |
| ANIMAL? | container |
|  |  |
| ANIMAL? | pencil |
|  |  |
| ANIMAL? | iguana |
|  |  |
| ANIMAL? | chisel |
|  |  |
| INACTION | raptor |
|  |  |
| INACTION | plate |
|  |  |
| ANIMAL? | shrimp |
|  |  |
| ANIMAL? | washing-machine |
|  |  |
| INACTION | syringe |
|  |  |
| ANIMAL? | lotto |
|  |  |
| ANIMAL? | sink |
|  |  |
| ANIMAL? | coat |
|  |  |
| INACTION | pillow |
|  |  |
| ANIMAL? | iceberg |
|  |  |
| INACTION | plow |
|  |  |
| INACTION | magnolia |
|  |  |
| ANIMAL? | perch |
|  |  |
| ANIMAL? | fortress |
|  |  |
| ANIMAL? | seagull |
|  |  |
| INACTION | weasel |
|  |  |
| INACTION | paddle |
|  |  |
| ANIMAL? | locker |
|  |  |
| ANIMAL? | mice |
|  |  |
| ANIMAL? | lion |
|  |  |
| INACTION | knife |
|  |  |
| INACTION | billy |
|  |  |
| ANIMAL? | bracelet |
|  |  |
| INACTION | spider |
|  |  |
| ANIMAL? | bell |
|  |  |
| INACTION | sheep |
|  |  |
| ANIMAL? | shark |
|  |  |
| INACTION | radio |
|  |  |
| ANIMAL? | gun |
|  |  |
| ANIMAL? | quail |
|  |  |
| INACTION | trout |
|  |  |
| ANIMAL? | notice |
|  |  |
| INACTION | mammoth |
|  |  |
| ANIMAL? | couch |
|  |  |
| INACTION | opossum |
|  |  |
| ANIMAL? | boat |
|  |  |
| INACTION | chimpanzee |
|  |  |
| INACTION | crawfish |
|  |  |
| ANIMAL? | cock |
|  |  |
| ANIMAL? | butterfly |
|  |  |
| INACTION | carnivore |
|  |  |
| ANIMAL? | seal |
|  |  |
| ANIMAL? | jenny |
|  |  |
| ANIMAL? | album |
|  |  |
| INACTION | gazelle |
|  |  |
| ANIMAL? | horse |
|  |  |
| ANIMAL? | moose |
|  |  |
| ANIMAL? | toad |
|  |  |
| ANIMAL? | racer |
|  |  |
| INACTION | train |
|  |  |
| ANIMAL? | revolver |
|  |  |
| INACTION | insect |
|  |  |
| ANIMAL? | ovals |
|  |  |
| INACTION | coffee pot |
|  |  |
| ANIMAL? | rhinoceros |
|  |  |
| ANIMAL? | eel |
|  |  |
| ANIMAL? | grasshopper |
|  |  |
| INACTION | kitty |
|  |  |
| INACTION | crutch |
|  |  |
| ANIMAL? | piano |
|  |  |
| ANIMAL? | baggage |
|  |  |
| ANIMAL? | tank |
|  |  |
| INACTION | crayfish |
|  |  |
| ANIMAL? | cheetah |
|  |  |
| ANIMAL? | penguin |
|  |  |
| ANIMAL? | boar |
|  |  |
| ANIMAL? | pony |
|  |  |
| ANIMAL? | blackbird |
|  |  |
| INACTION | partridge |
|  |  |
| INACTION | bison |
|  |  |
| INACTION | bear |
|  |  |
| ANIMAL? | ashtray |
|  |  |
| ANIMAL? | washbasin |
|  |  |
| ANIMAL? | mare |
|  |  |
| ANIMAL? | soap |
|  |  |
| ANIMAL? | ring |
|  |  |
| INACTION | locust |
|  |  |
| INACTION | rectangle |
|  |  |
| ANIMAL? | turkey |
|  |  |
| ANIMAL? | accordion |
|  |  |
| ANIMAL? | clarinet |
|  |  |
| ANIMAL? | plaster |
|  |  |
| ANIMAL? | sponge |
|  |  |
| ANIMAL? | bull |
|  |  |
| INACTION | mammal |
|  |  |
| INACTION | robin |
|  |  |
| ANIMAL? | tiger |
|  |  |
| INACTION | clinic |
|  |  |
| ANIMAL? | fence |
|  |  |
| ANIMAL? | microphone |
|  |  |
| ANIMAL? | camping |
|  |  |
| INACTION | shovel |
|  |  |
| INACTION | target |
|  |  |
| ANIMAL? | veal |
|  |  |
| ANIMAL? | bowl |
|  |  |
| ANIMAL? | broom |
|  |  |
| ANIMAL? | headband |
|  |  |
| INACTION | nightingale |
|  |  |
| ANIMAL? | hamster |
|  |  |
| ANIMAL? | axe |
|  |  |
| INACTION | accommodation |
|  |  |
| ANIMAL? | case |
|  |  |
| ANIMAL? | bump |
|  |  |
| INACTION | dome |
|  |  |
| ANIMAL? | doe |
|  |  |
| ANIMAL? | feline |
|  |  |
| ANIMAL? | vulture |
|  |  |
| ANIMAL? | cork |
|  |  |
| ANIMAL? | scorpion |
|  |  |
| INACTION | lamppost |
|  |  |
| ANIMAL? | chameleon |
|  |  |
| ANIMAL? | umbrella |
|  |  |
| INACTION | caterpillar |
|  |  |
| ANIMAL? | rifle |
|  |  |
| INACTION | cover |
|  |  |
| ANIMAL? | Dog |
|  |  |
| INACTION | checkbook |
|  |  |
| ANIMAL? | spool |
|  |  |
| ANIMAL? | frog |
|  |  |
| INACTION | fly |
|  |  |
| ANIMAL? | sweater |
|  |  |
| ANIMAL? | glue |
|  |  |
| ANIMAL? | flute |
|  |  |
| ANIMAL? | bottle |
|  |  |
| ANIMAL? | caribou |
|  |  |
| ANIMAL? | canapy |
|  |  |
| ANIMAL? | grizzly |
|  |  |
| INACTION | piston |
|  |  |
| INACTION | squirrel |
|  |  |
| ANIMAL? | ox |
|  |  |
| ANIMAL? | string |
|  |  |
| ANIMAL? | budgie |
|  |  |
| ANIMAL? | cow |
|  |  |
| INACTION | slipper |
|  |  |
| ANIMAL? | casserole |
|  |  |
| INACTION | bird |
|  |  |
| ANIMAL? | cauldron |
|  |  |
| INACTION | salmon |
|  |  |
| INACTION | sardine |
|  |  |
| INACTION | racket |
|  |  |
| ANIMAL? | dolphin |
|  |  |
| ANIMAL? | ferret |
|  |  |
| INACTION | beluga |
|  |  |
| INACTION | shearer |
|  |  |
| ANIMAL? | dalmatian |
|  |  |
| ANIMAL? | koala |
|  |  |
| ANIMAL? | dagger |
|  |  |
| ANIMAL? | owl |
|  |  |
| INACTION | ewe |
|  |  |
| ANIMAL? | hare |
|  |  |
| ANIMAL? | squid |
|  |  |
| ANIMAL? | cricket |
|  |  |
| ANIMAL? | gorilla |
|  |  |
| ANIMAL? | coyote |
|  |  |
| ANIMAL? | stopwatch |
|  |  |
| ANIMAL? | ostrich |
|  |  |
| INACTION | quadrate |
|  |  |
| ANIMAL? | stool |
|  |  |
| INACTION | crow |
|  |  |
| INACTION | glove |
|  |  |
| INACTION | dormitory |
|  |  |
| ANIMAL? | parcel |
|  |  |
| ANIMAL? | snail |
|  |  |
| INACTION | canary |
|  |  |
| ANIMAL? | rat |
|  |  |
| ANIMAL? | stagecoach |
|  |  |
| ANIMAL? | raven |
|  |  |
| ANIMAL? | goat |
|  |  |
| ANIMAL? | avalanche |
|  |  |
| ANIMAL? | polish |
|  |  |
| ANIMAL? | skate |
|  |  |
| ANIMAL? | drawer |
|  |  |
| ANIMAL? | donkey |
|  |  |
| ANIMAL? | dryer |
|  |  |
| ANIMAL? | trash |
|  |  |
| ANIMAL? | faucet |
|  |  |
| INACTION | mole |
|  |  |
| ANIMAL? | peaches |
|  |  |
| ANIMAL? | cannon |
|  |  |
| ANIMAL? | goose |
|  |  |
| ANIMAL? | skunk |
|  |  |
| ANIMAL? | petticoat |
|  |  |
| ANIMAL? | pike |
|  |  |
| INACTION | tire |
|  |  |
| ANIMAL? | computer |
|  |  |
| ANIMAL? | archive |
|  |  |
| ANIMAL? | roller |
|  |  |
| ANIMAL? | compass |
|  |  |
| INACTION | poodle |
|  |  |
| INACTION | review |
|  |  |
| INACTION | vase |
|  |  |
| INACTION | cupboard |
|  |  |
| ANIMAL? | castle |
|  |  |
| ANIMAL? | foal |
|  |  |
| ANIMAL? | pen |
|  |  |
| ANIMAL? | beret |
|  |  |
| ANIMAL? | snake |
|  |  |
| INACTION | chamois |
|  |  |
| INACTION | pelican |
|  |  |
| INACTION | beaver |
|  |  |
| ANIMAL? | oyster |
|  |  |
| INACTION | dynamite |
|  |  |
| ANIMAL? | stallion |
|  |  |
| ANIMAL? | screwdriver |
|  |  |
| ANIMAL? | lark |
|  |  |
| ANIMAL? | dove |
|  |  |
| INACTION | jewel |
|  |  |
| ANIMAL? | flag |
|  |  |
| ANIMAL? | ape |
|  |  |
| ANIMAL? | post |
|  |  |
| INACTION | pig |
|  |  |
| INACTION | ticket |
|  |  |
| INACTION | propeller |
|  |  |
| ANIMAL? | saw |
|  |  |
| ANIMAL? | dinosaur |
|  |  |
| ANIMAL? | bee |
|  |  |
| ANIMAL? | beetle |
|  |  |
| INACTION | giraffe |
|  |  |
| ANIMAL? | pyramid |
|  |  |
| ANIMAL? | jar |
|  |  |
| INACTION | fawn |
|  |  |
| INACTION | fork |
|  |  |
| ANIMAL? | tissue |
|  |  |
| ANIMAL? | herbivore |
|  |  |
| INACTION | chest |
|  |  |
| ANIMAL? | crocodile |
|  |  |
| INACTION | kettle |
|  |  |
| INACTION | elephant |
|  |  |
| ANIMAL? | cougar |
|  |  |
| ANIMAL? | ball |
|  |  |
| ANIMAL? | fox |
|  |  |
| ANIMAL? | cobra |
|  |  |
| ANIMAL? | aphid |
|  |  |
| ANIMAL? | hyena |
|  |  |
| ANIMAL? | buffalo |
|  |  |
| INACTION | mosquito |
|  |  |
| ANIMAL? | falcon |
|  |  |
| ANIMAL? | lama |
|  |  |
| ANIMAL? | calendar |
|  |  |
| ANIMAL? | label |
|  |  |
| INACTION | pool |
|  |  |
| INACTION | crab |
|  |  |
| INACTION | sparrow |
|  |  |
| INACTION | peacock |
|  |  |
| INACTION | cassette |
|  |  |
| INACTION | osprey |
|  |  |
| ANIMAL? | dragonfly |
|  |  |
| INACTION | lobster |
|  |  |
| ANIMAL? | hippopotamus |
|  |  |
| ANIMAL? | labrador |
|  |  |
| ANIMAL? | wolf |
|  |  |
| ANIMAL? | passport |
|  |  |
| ANIMAL? | dish |
|  |  |
| ANIMAL? | violin |
|  |  |
| ANIMAL? | shoe |
|  |  |
| ANIMAL? | garbage |
|  |  |
| ANIMAL? | swan |
|  |  |
| ANIMAL? | hat |
|  |  |
| INACTION | chicken |
|  |  |
| INACTION | key |
|  |  |
| ANIMAL? | tuna |
|  |  |
| ANIMAL? | cat |
|  |  |
| INACTION | jackal |
|  |  |
| ANIMAL? | rodent |
|  |  |
| ANIMAL? | mirror |
|  |  |
| ANIMAL? | comb |
|  |  |
| INACTION | circuit |
|  |  |
| INACTION | ant |
|  |  |
| ANIMAL? | seesaw |
|  |  |
| ANIMAL? | python |
|  |  |
| INACTION | kangaroo |
|  |  |
| INACTION | plane |
|  |  |
| INACTION | towel |
|  |  |
| INACTION | drill |
|  |  |
| ANIMAL? | squab |
|  |  |
| INACTION | padlock |
|  |  |
| ANIMAL? | fertilizer |
|  |  |
| ANIMAL? | sprinkler |
|  |  |
| ANIMAL? | pane |
|  |  |
| INACTION | hoop |
|  |  |
| ANIMAL? | turtle |
|  |  |
| ANIMAL? | lynx |
|  |  |
| ANIMAL? | airport |
|  |  |
| ANIMAL? | zebra |
|  |  |
| ANIMAL? | otter |
|  |  |
| INACTION | van |
|  |  |
| INACTION | ram |
|  |  |
| ANIMAL? | flake |
|  |  |
| ANIMAL? | stapler |
|  |  |
| ANIMAL? | shirt |
|  |  |
| ANIMAL? | cave |
|  |  |
| ANIMAL? | mummy |
|  |  |
| INACTION | necklace |
|  |  |
| ANIMAL? | ladybug |
|  |  |
| INACTION | sow |
